# Association of body mass index with postoperative pulmonary complications and potential explanatory pathways in paediatric scoliosis correction surgery: a multicentre cohort study

**DOI:** 10.1080/07853890.2026.2731716

**Published:** 2026-09-17

**Authors:** Weihai Shi, Shouqiang Zhu, Yan Wang, Yanhua Sun, Zhiying Zheng, Peiyu Liu, Ruocui Zhang, Tianjiao Xia, Lulu Ma, Min Li, Jinhua Bo, Xiaoping Gu

**Affiliations:** ^a^ Department of Anesthesiology, Nanjing Drum Tower Hospital, Affiliated Hospital of Medical School, Nanjing University, Nanjing, China; ^b^ Medical School of Nanjing University, Nanjing, China; ^c^ Drum Tower Clinical Medical School, Nanjing Medical University, Nanjing, China; ^d^ Department of Operating Room, Nanjing Drum Tower Hospital, Affiliated Hospital of Medical School, Nanjing University, Nanjing, China; ^e^ Post-Anesthesia Care Unit, Department of Anesthesiology and Surgery, Nanjing Drum Tower Hospital, Nanjing, China; ^f^ Department of Anesthesiology, Peking Union Medical College Hospital, Beijing, China; ^g^ Department of Anesthesiology, Peking University Third Hospital, Beijing, China

**Keywords:** Body mass index, paediatric scoliosis, scoliosis surgery, postoperative pulmonary complications

## Abstract

**Background:**

Postoperative pulmonary complications (PPCs) contribute substantially to morbidity after paediatric scoliosis correction surgery, but the relationship between preoperative body mass index (BMI) and PPCs remains uncertain. We evaluated this association and explored potential explanatory pathways and the incremental predictive value of BMI.

**Methods:**

This retrospective cohort study included 1,165 patients from three Chinese tertiary hospitals. Associations between BMI and the composite PPC outcome were evaluated using multivariable regression. An orthogonalized causal forest estimated the average partial effect of continuous BMI on PPC probability, and variation in model-derived partial-effect predictions across sex and preoperative Cobb-angle strata was examined descriptively. Exploratory pathway analyses considered the Prognostic Nutritional Index (PNI), intraoperative blood loss, and fusion levels. At the completion of surgery, the incremental predictive value of BMI was assessed using an elastic-net model developed in centres 1 and 3 and externally validated in centre 2.

**Results:**

PPCs occurred in 321 patients (27.55%). In the primary preoperative-adjusted model, each 1-kg/m^2^ higher BMI was associated with lower odds of PPCs (aOR, 0.87; 95% CI, 0.83–0.91; *p* < 0.001). A secondary exploratory perioperative-adjusted model yielded a similar estimate (aOR, 0.85; 95% CI, 0.81–0.89; *p* < 0.001). Compared with normal weight, thinness was associated with higher odds of PPCs (aOR, 4.26; 95% CI, 2.74–6.67; *p* < 0.001), whereas overweight was associated with lower odds (aOR, 0.57; 95% CI, 0.34–0.91; *p* = 0.022). The causal forest yielded an inverse average partial-effect estimate for BMI (−0.031; 95% CI, −0.037 to −0.025; *p* < 0.001). Exploratory pathway analyses suggested small indirect components through PNI, blood loss, and fusion levels. Adding BMI increased the external-validation AUC from 0.658 to 0.703 (*p* < 0.001), representing a modest improvement in discrimination.

**Conclusions:**

Higher preoperative BMI was associated with lower odds of PPCs, with a concordant inverse causal-forest estimate under its identification assumptions. Low BMI or thinness may serve as a preoperative risk marker, while the examined pathways accounted for only small proportions of the association. At the end-of-surgery prediction time point, adding BMI provided modest incremental predictive value, although the model’s external performance was modest and further prospective validation is required.

## Graphical Abstract

**Figure:**
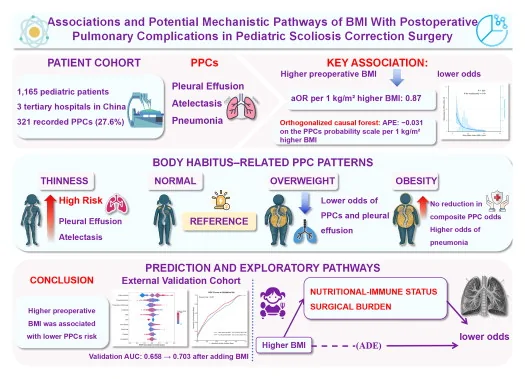

## Introduction

Postoperative pulmonary complications (PPCs) remain a primary driver of perioperative morbidity and extended hospital stays following paediatric scoliosis correction surgery, contributing significantly to the clinical and economic burden [1]. The physiological impact of extensive spinal fusion is profound, particularly in the paediatric population where incomplete respiratory development often coexists with restrictive lung disease secondary to chest wall deformity. The substantial metabolic stress, prolonged operative duration, and significant blood loss associated with these procedures further predispose these vulnerable patients to respiratory failure, atelectasis, and pneumonia [2]. Consequently, precise risk stratification is essential for optimizing perioperative management and improving surgical outcomes.

The impact of body mass index (BMI) and body habitus on surgical prognosis has been increasingly recognized as complex. While obesity has traditionally been associated with technical challenges and complications such as surgical site infections [3,4], the ‘obesity paradox’ observed in various surgical cohorts suggests that overweight or mildly obese individuals may exhibit superior resilience to metabolic stress compared to their underweight peers [5–7]. In the context of paediatric scoliosis, this relationship is further complicated by the potential for ‘pseudobesity’ or severe malnutrition masked by spinal deformity. Current evidence remains conflicting: some studies have associated obesity with greater resource utilization [8], while others identify low BMI as an independent predictor of adverse outcomes including pneumonia and readmission [9]. These inconsistencies suggest that the influence of BMI may be heterogeneous, varying with surgical complexity and patient-specific physiological reserve [10,11].

Furthermore, most existing studies have relied on conventional association models and have not examined the BMI–PPC relationship using flexible causal-inference methods. Using a multicentre cohort, we aimed to evaluate the association between preoperative BMI and PPCs, examine its consistency within an orthogonalized causal-forest framework, explore potential explanatory pathways, and assess the incremental predictive value of BMI.

## Methods

This multicentre retrospective cohort study included patients treated between January 1, 2018, and December 31, 2024, at three tertiary hospitals in China: Nanjing Drum Tower Hospital, Affiliated Hospital of Medical School, Nanjing University (centre 1); Peking University Third Hospital (centre 2); and Peking Union Medical College Hospital (centre 3). Clinical data were extracted from the electronic medical record and perioperative management systems of the participating hospitals. The study was conducted in accordance with the Declaration of Helsinki and was approved by the Institutional Review Board of Nanjing Drum Tower Hospital, Affiliated Hospital of Medical School, Nanjing University (approval no. 2026-0261-01). The approval covered the analysis of the anonymized multicentre dataset, and the requirement for informed consent was waived because of the retrospective design and use of de-identified data. The study was reported in accordance with the STROBE statement [12].

We included patients aged <18 years who underwent primary scoliosis correction surgery. We excluded patients with a history of respiratory failure, pneumonia, pleural effusion, atelectasis, acute respiratory distress syndrome, or pneumothorax within the past year; those with missing data on preoperative BMI; or those with an American Society of Anesthesiologists (ASA) physical status classification of >4. Because a patient’s height may change after scoliosis correction surgery, thereby altering BMI, preoperative BMI was used as the exposure variable and was defined as the value calculated from the height and weight measurements recorded closest to the date of surgery. Preoperative height and weight were obtained from routine medical records as part of standard preoperative assessment, with height generally measured without shoes using a stadiometer and weight measured without shoes using a calibrated scale. BMI was calculated as weight divided by height squared (kg/m^2^) and was analysed as a continuous variable. To address the potential influence of scoliosis-related height distortion on BMI estimation, we performed a supplementary logistic regression analysis using preoperative body weight as an alternative exposure. The same hierarchical adjustment strategy was applied where appropriate. The BMI-Z score, a statistical measure based on the World Health Organization (WHO) child growth standards, was used to define body habitus [13]. Children were categorized into four mutually exclusive body-habitus groups according to BMI-Z score: thinness (low BMI-Z, <−1), normal BMI-Z (−1 to 1), overweight (>1 to ≤2), and obesity (>2). These categories were used to examine the association between body habitus and PPCs. Postoperative pulmonary complications occurring within 7 days after surgery were categorized with reference to the European Perioperative Clinical Outcome (EPCO) definitions and included respiratory failure, pneumonia, pleural effusion, atelectasis, bronchospasm, acute respiratory distress syndrome, and pneumothorax [14,15]. Outcome ascertainment relied on retrospectively documented postoperative diagnoses and available imaging reports, including chest computed tomography reports when imaging was performed as part of routine clinical care. Postoperative imaging was obtained at the discretion of the treating clinicians and was not governed by a standardized prospective imaging protocol across the participating centres. Because the source databases did not consistently contain all of the clinical, physiological, and imaging details required for component-specific EPCO adjudication, it was not possible to independently re-adjudicate every recorded event according to the complete EPCO diagnostic criteria. The outcome should therefore be interpreted as a retrospectively recorded, EPCO-informed composite rather than a prospectively standardized or independently adjudicated EPCO endpoint. Patients with one or more recorded PPCs were counted once in the composite outcome.

### Missing data

The amount and percentage of missingness for each variable are summarized in Tables S1 and S^2^. Missing data were handled using multiple imputation by chained equations under the missing-at-random assumption. The imputation model included all variables used in the main analyses. PPC status and study center were included as auxiliary predictors in the imputation model to preserve their associations with other variables, but they were not imputed. A total of 10 imputed datasets were generated, and estimates were pooled using Rubin’s rules. Complete-case analyses were also performed as sensitivity analyses.

**Table 1.** Baseline clinical Characteristics of patients stratified by PPCs.

Variables	Total (*n* = 1,165)	Without PPCs (*n* = 844)	With PPCs (*n* = 321)	*P*
BMI (kg/m²)	18.30 (16.79, 20.71)	18.78 (17.01, 21.52)	17.44 (15.98, 19.26)	<0.001
Sex, n (%)				<0.001
Female children	858 (73.65)	595 (70.50)	263 (81.93)	
Male children	307 (26.35)	249 (29.50)	58 (18.07)	
Body habitus, n (%)				<0.001
Normal weight	886 (76.05)	658 (77.96)	228 (71.03)	
Obesity	24 (2.06)	15 (1.78)	9 (2.80)	
Overweight	149 (12.79)	125 (14.81)	24 (7.48)	
Thinness	106 (9.10)	46 (5.45)	60 (18.69)	
Age (years)	14 (13, 15)	14 (13, 15)	14 (12, 15)	0.013
ALT (U/L)	10.00 (8.00, 13.80)	10.00 (8.00, 13.00)	10.60 (8.20, 14.10)	0.129
AST (U/L)	18.00 (16.00, 20.70)	18.00 (16.00, 20.00)	18.20 (16.00, 22.00)	0.078
Albumin (g/L)	43.50 (41.50, 45.80)	43.80 (41.80, 46.00)	42.90 (40.90, 45.40)	0.003
Total Bilirubin (μmol/L)	10.50 (7.80, 14.10)	10.60 (7.80, 13.90)	10.50 (7.60, 14.70)	0.617
Creatinine (μmol/L)	51.00 (44.00, 59.00)	52.00 (45.00, 60.00)	48.00 (41.00, 56.00)	<0.001
WBC (×10⁹/L)	6.37 (5.44, 7.38)	6.40 (5.46, 7.40)	6.30 (5.40, 7.30)	0.550
Lymphocyte (×10⁹/L)	2.43 (1.98, 2.90)	2.50 (2.03, 2.90)	2.32 (1.85, 2.80)	0.003
Hemoglobin (g/L)	131.00 (124.00, 141.00)	132.00 (123.00, 142.00)	131.00 (124.00, 136.00)	0.002
Platelets (×10⁹/L)	259.00 (222.00, 305.00)	260.00 (225.00, 307.25)	256.00 (220.00, 297.00)	0.103
Fibrinogen (g/L)	2.30 (1.98, 2.69)	2.29 (1.96, 2.69)	2.37 (2.10, 2.70)	0.326
Prothrombin Time (s)	11.60 (11.10, 12.10)	11.60 (11.20, 12.22)	11.40 (11.00, 12.00)	<0.001
APTT (s)	29.50 (27.70, 31.80)	29.60 (27.80, 31.70)	29.20 (27.30, 31.90)	0.060
Blood Loss (mL)	600.00 (400.00, 800.00)	500.00 (327.75, 800.00)	700.00 (500.00, 1000.00)	<0.001
Urine Output (mL)	900.00 (550.00, 1300.00)	800.00 (500.00, 1200.00)	1000.00 (700.00, 1400.00)	<0.001
Crystalloid Infusion (mL)	1800.00 (1300.00, 2400.00)	1900.00 (1445.00, 2500.00)	1700.00 (1100.00, 2200.00)	<0.001
Colloid Infusion (mL)	1000.00 (0.00, 1500.00)	500.00 (0.00, 1100.00)	1500.00 (500.00, 1500.00)	<0.001
Number of fusion levels	10.00 (8.00, 12.00)	9.00 (7.00, 12.00)	10.00 (9.00, 12.00)	<0.001
Preoperative Cobb Angle (°)	54.00 (45.00, 64.00)	51.00 (43.00, 62.00)	58.00 (50.00, 70.00)	<0.001
Operative Time (min)	245.00 (201.60, 303.00)	244.80 (198.00, 300.00)	259.20 (216.00, 331.20)	<0.001
Postoperative Hospital Stay (days)	8 (6, 9)	8 (6, 9)	8.00 (7, 9)	0.852
Total Hospital Stay (days)	14 (10, 16)	14 (10, 16)	14 (11, 16)	0.283

Data are presented as median (IQR) for continuous variables and n (%) for categorical variables. Abbreviations: ALT, alanine aminotransferase; APTT, activated partial thromboplastin time; AST, aspartate aminotransferase; BMI, body mass index; IQR, interquartile range; PPCs, postoperative pulmonary complications; WBC, white blood cell count.

### Statistical analysis

We constructed a directed acyclic graph to conceptualize the relationship between the exposure and the outcome (Figure S1). The primary preoperative adjustment set included sex, age, alanine aminotransferase (ALT), aspartate aminotransferase (AST), albumin, creatinine, white blood cell count (WBC), haemoglobin, platelets, fibrinogen, preoperative Cobb angle, and study centre. Perioperative variables, including blood loss, urine output, crystalloid and colloid infusion, fusion levels, and operative time, were considered potential intermediate factors or markers of surgical complexity and were included only in the secondary exploratory model. Continuous variables were summarized as mean (standard deviation [SD]) or median (interquartile range [IQR]) and compared using the two-sample t-test or Mann–Whitney U-test, as appropriate; normality was assessed using the Kolmogorov–Smirnov test. Categorical data were expressed as counts (percentages) and compared using the chi-square test or Fisher’s exact test. Univariable and multivariable logistic regression analyses were used to examine the relationship between BMI, body habitus, and PPCs. Model 1 included only BMI. Model 2 was adjusted for sex, age, and study centre. Model 3 was designated as the primary preoperative-adjusted model and included sex, age, ALT, AST, albumin, creatinine, WBC, haemoglobin, platelets, fibrinogen, preoperative Cobb angle, and study centre. This model was used to estimate the total association between preoperative BMI and PPCs. Model 4 additionally included blood loss, urine output, crystalloid and colloid infusion, fusion levels, and operative time and was considered a secondary exploratory perioperative-adjusted model. These perioperative variables were not regarded as conventional confounders because they may reflect surgical complexity, intraoperative management, or intermediate pathways. Therefore, models including these intraoperative variables were interpreted as exploratory perioperative-adjusted models rather than primary total-effect models. The relationship between body habitus (based on BMI-Z score) and three common pulmonary complications—pleural effusion, pneumonia, and atelectasis—was also investigated. The association between continuous BMI and PPCs was additionally examined using a restricted cubic spline logistic regression model with 4 knots. The model used the same covariates as the primary preoperative-adjusted model. Overall association and nonlinearity were evaluated using Wald tests.

### Causal-forest analysis

Causal-forest analyses were conducted using the grf package in R. BMI was modelled as a continuous exposure and PPC status as a binary outcome. The covariate matrix was restricted to pre-exposure variables and included sex, age, ALT, AST, albumin, creatinine, WBC, haemoglobin, platelets, fibrinogen, preoperative Cobb angle, and study centre. Preoperative body weight was not included in the causal-forest covariate matrix, and BMI and body weight were not entered simultaneously in any causal-forest model. Body weight was examined only as an alternative exposure in a separate supplementary regression analysis. An orthogonalized causal forest was fitted with 2,000 trees using tune.parameters = ‘all’ and a prespecified random seed of 123. Exposure and outcome nuisance functions were estimated internally using regression forests and out-of-bag predictions [16]. The causal-forest analysis was performed separately in each of the 10 imputed datasets, with all 1,165 patients contributing to each analysis; the resulting estimates and variances were combined using Rubin’s rules. Thus, the primary causal-forest analysis was not restricted to complete cases.

Because BMI was modelled as a continuous exposure, the estimand obtained using average_treatment_effect() was interpreted as the average partial effect, E{Cov(BMI, PPCs | X)/Var(BMI | X)}, rather than a conventional average treatment effect for a binary exposure. On the binary-outcome probability scale, this estimand represents a model-based average conditional linear slope per 1 kg/m^2^ higher BMI. Within each imputed dataset, the overall average partial effect and its variance were obtained using the doubly robust procedure implemented in average_treatment_effect(). Estimates were subsequently combined across the 10 imputed datasets using Rubin’s rules, incorporating both within- and between-imputation variance [17].

Empirical support for continuous BMI was assessed across sex, study centre, and preoperative Cobb-angle strata. Within each stratum, we summarized the observed BMI range, the 1st–99th percentile interval, and the number and proportion of patients within the central common-support interval. For each stratification variable, the central common-support interval was defined as the intersection of the subgroup-specific 1st–99th percentile intervals. Kernel density curves were used to visualize the BMI distribution across strata, and the distribution of model-derived conditional partial-effect predictions was examined descriptively.

### Exploratory pathway analysis

Exploratory pathway analyses were performed to examine whether nutritional-immune status and operative burden might represent potential indirect components of the association between BMI and PPCs. The nutritional-immune pathway was represented by the Prognostic Nutritional Index (PNI), calculated as albumin (g/L) + 5 × lymphocyte count (×10^9^/L), whereas operative burden was represented by intraoperative blood loss and the number of fusion levels. The mediation models generated quantities conventionally termed the average causal mediation effect (ACME) and average direct effect (ADE); however, in this retrospective analysis, these estimates were interpreted only as model-based indirect and remaining (direct) components of the observed association, rather than as confirmed causal effects. All models were adjusted for sex, age, ALT, AST, creatinine, WBC, haemoglobin, platelets, fibrinogen, preoperative Cobb angle, and study centre. Estimates were obtained using quasi-Bayesian Monte Carlo simulation with 1,000 iterations. The estimated proportion attributed to each pathway was calculated as the ratio of the ACME to the estimated total association. Because the temporal ordering and assumptions required for causal mediation could not be fully established, all pathway analyses were considered exploratory and hypothesis-generating. Albumin was not additionally included in the PNI model because it is a component of PNI.

### Predictive value

The prediction time point was defined as completion of surgery, when all intraoperative predictors were available and before ascertainment of PPCs. The development cohort comprised patients from centres 1 and 3 (*n* = 882; 243 PPC events), and centre 2 served as the external validation cohort (*n* = 283; 78 PPC events). Two elastic-net logistic regression models were developed: a base model including sex, age, ALT, AST, albumin, creatinine, WBC, haemoglobin, platelets, fibrinogen, blood loss, urine output, crystalloid and colloid infusion, fusion levels, preoperative Cobb angle, and operative time, and a full model additionally including BMI. Study centre was not included as a predictor because it defined the geographic development–validation split.

The cohorts were separated before missing-data handling. Ten datasets were generated separately for each cohort using multiple imputation by chained equations. PPC status was included only as an auxiliary variable in the development-cohort imputation model and was neither imputed nor used to impute predictors in the validation cohort. The 10 imputed development datasets were stacked, with each record assigned a weight of 1/10. During 5-fold cross-validation, all imputed copies of the same patient were retained within the same fold, and the elastic-net parameters were selected using minimum cross-validated deviance. The resulting models were applied unchanged to each imputed validation dataset, without further variable selection, parameter tuning, or recalibration. Patient-level predicted probabilities were subsequently averaged across the 10 imputations.

Discrimination was assessed using the area under the receiver operating characteristic curve (AUC) with 95% CIs, and differences in AUC were compared using the DeLong test. Calibration was evaluated using calibration plots, calibration intercepts and slopes, and Brier scores. Incremental predictive performance was further assessed using the net reclassification improvement (NRI), integrated discrimination improvement (IDI), and decision curve analysis (DCA). Sensitivity and specificity in the validation cohort were calculated using the corresponding development-cohort thresholds determined by the Youden index. Complete predictor coding, model intercepts, coefficients, and selected tuning parameters are provided in Table S3. SHapley Additive exPlanations (SHAP) was used to illustrate predictor contributions to the full-model output. The prediction-model analysis was reported in accordance with the Transparent Reporting of a multivariable prediction model for Individual Prognosis Or Diagnosis (TRIPOD) statement [18]. Statistical analyses were performed using R version 4.4.1.

### Sensitivity analysis

A sensitivity analysis was performed using a complete-case approach to evaluate the robustness of our primary findings, excluding patients with missing baseline or intraoperative data. We constructed four hierarchical logistic regression models using the same adjustment hierarchy as in the main analysis. Model 1 was an unadjusted (crude) model. Model 2 was adjusted for demographic factors (sex and age). Model 3 was designated as the primary preoperative-adjusted model and additionally included preoperative laboratory parameters (ALT, AST, albumin, creatinine, WBC, haemoglobin, platelets, and fibrinogen) and preoperative Cobb angle. Model 4 was designated as a secondary exploratory perioperative-adjusted model and additionally included blood loss, urine output, crystalloid and colloid infusion, fusion levels, and operative time. The perioperative variables in Model 4 were considered potential intermediate factors or markers of surgical complexity rather than conventional confounders. To account for potential centre-specific effects, Models 2–4 included study centre as a fixed effect.

## Results

### Baseline characteristics

**Figure 1.**
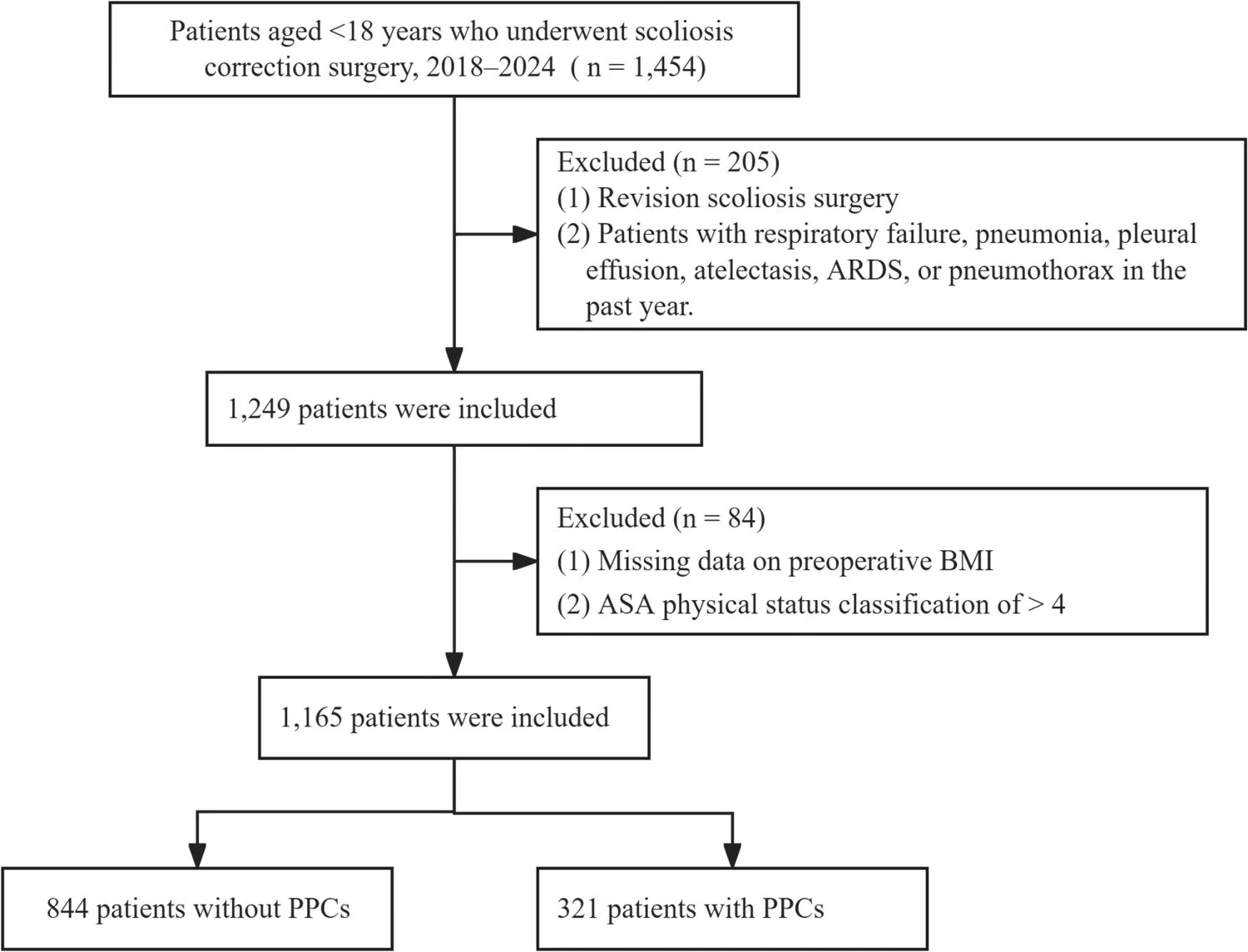
Study flow diagram.

A total of 1,165 paediatric patients who underwent scoliosis correction surgery across three centres were included, of whom 321 (27.55%) developed PPCs (Figure 1). Among the 321 patients with PPCs, subtype frequencies were summarized using mutually exclusive categories based on the primary documented postoperative diagnosis. Pleural effusion was the primary documented PPC category in 213 patients (66.36%), followed by pneumonia in 59 (18.38%), atelectasis in 43 (13.40%), pneumothorax in 4 (1.25%), and respiratory failure in 2 (0.62%) (Tables S4 and S^5^). Compared with patients without PPCs, patients in the PPC group were younger (*p* = 0.013) and had a higher proportion of females (81.93% vs. 70.50%, *p* < 0.001). Regarding the primary exposure, baseline BMI was significantly lower in the PPC group (17.44 vs. 18.78 kg/m^2^, *p* < 0.001). This pattern was consistent with the body-habitus categories, as the PPC group showed a higher prevalence of thinness (18.69% vs. 5.45%) and a lower prevalence of overweight (7.48% vs. 14.81%) (*p* < 0.001). Furthermore, the PPC group exhibited lower preoperative levels of albumin, creatinine, and hemoglobin, alongside a shorter prothrombin time (all *p* < 0.05). Surgical characteristics indicated greater operative complexity in patients with PPCs, characterized by larger preoperative Cobb angles (58.00° vs. 51.00°, *p* < 0.001), more fusion levels (*p* < 0.001), and prolonged operative times (259.2 vs. 244.8 min, *p* < 0.001). They also had greater blood loss and urine output and received larger volumes of colloid infusion but smaller volumes of crystalloid infusion (Table 1). Variable-specific missingness patterns according to study centre and PPC status are reported in Table S2. No clear differences in missingness were observed according to PPC status. Nevertheless, study centre and PPC status were included as auxiliary predictors in the multiple-imputation model to preserve their associations with the imputed variables.

### Association between BMI and PPCs

**Figure 2.**
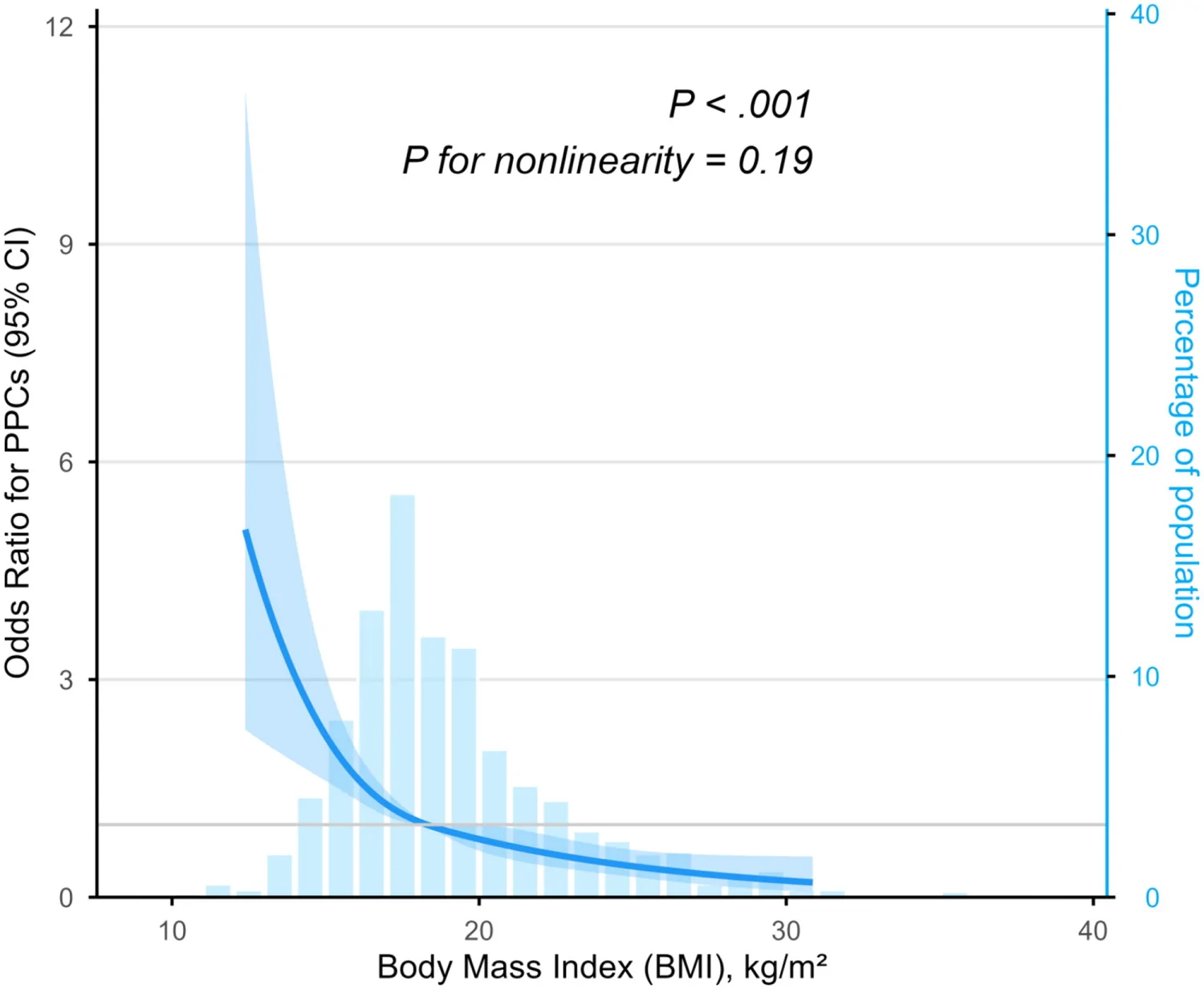
Dose-response association between BMI and the odds of PPCs. Restricted cubic spline (RCS) analysis of the dose-response relationship between BMI and the risk of PPCs in paediatric scoliosis surgery. The solid blue line represents the aOR, and the light blue shaded area denotes the 95% CI. The background histogram illustrates the percentage distribution of BMI within the study population (right y-axis). The model was adjusted for prespecified preoperative covariates, including sex, age, ALT, AST, albumin, creatinine, WBC, haemoglobin, platelets, fibrinogen, preoperative Cobb angle, and study centre. *P* < 0.001 for the overall association and P = 0.187 for nonlinearity.

Multivariable logistic regression models were constructed to evaluate the adjusted association between BMI and the odds of PPCs. When assessed as a continuous variable, higher preoperative BMI was consistently associated with lower odds of PPCs across all models. In the primary preoperative-adjusted model, each 1 kg/m^2^ higher BMI was associated with 13% lower odds of PPCs (aOR, 0.87; 95% CI, 0.83–0.91; *p* < 0.001). The association remained similar in the secondary exploratory perioperative-adjusted model (aOR, 0.85; 95% CI, 0.81–0.89; *p* < 0.001) (Table 2 and Table S6). Furthermore, restricted cubic spline (RCS) analysis was performed to visualize the dose-response relationship between BMI and the risk of PPCs. The resulting curve suggested a significant overall association between BMI and PPCs (*p* < 0.001 for the overall association), with no evidence of nonlinearity (P for nonlinearity = 0.187; Figure 2). To further elucidate this relationship, patients were stratified by body habitus. In the categorical analysis using the same primary preoperative adjustment set and normal weight as the reference, thinness was associated with higher odds of PPCs (aOR, 4.26; 95% CI, 2.74–6.67; *p* < 0.001), whereas overweight was associated with lower odds (aOR, 0.57; 95% CI, 0.34–0.91; *p* = 0.022). Obesity was not significantly associated with overall PPCs (aOR, 1.56; 95% CI, 0.60–3.82; *p* = 0.341; Table S7).

**Table 2.** Association between preoperative BMI and PPCs in logistic regression models.

Variables	Model 1		Model 2		Model 3		Model 4	
	OR (95% CI)	*P*	aOR (95% CI)	*P*	aOR (95% CI)	*P*	aOR (95% CI)	*P*
BMI	0.86 (0.82, 0.90)	<0.001	0.87 (0.83, 0.91)	<0.001	0.87 (0.83, 0.91)	<0.001	0.85 (0.81, 0.89)	<0.001

Abbreviations: BMI, body mass index; PPCs, postoperative pulmonary complications; OR, odds ratio; aOR, adjusted odds ratio; CI, confidence interval.

Model 1: Crude.

Model 2: Adjust: Sex, Age.

Model 3 (Primary preoperative-adjusted model): Adjust: Sex, Age, ALT, AST, Albumin, Creatinine, WBC, Hemoglobin, Platelets, Fibrinogen, Preoperative Cobb Angle.

Model 4 (secondary exploratory perioperative-adjusted model): Adjust: Sex, Age, ALT, AST, Albumin, Creatinine, WBC, Hemoglobin, Platelets, Fibrinogen, Preoperative Cobb Angle, Blood Loss, Urine Output, Crystalloid Infusion, Colloid Infusion, Fusion Levels, Operative Time.

Models 2–4 included study center as a fixed effect in addition to the listed variables. Perioperative variables included in Model 4 were considered potential intermediate factors or markers of surgical complexity rather than conventional confounders.

In analyses of specific PPC subtypes, thinness was associated with higher odds of pleural effusion (aOR, 4.23; 95% CI, 2.72–6.60; *p* < 0.001) and atelectasis (aOR, 4.72; 95% CI, 1.95–10.48; *p* = 0.001). Obesity was associated with higher odds of postoperative pneumonia (aOR, 4.50; 95% CI, 1.34–12.54; *p* = 0.018). Conversely, overweight was associated with lower odds of pleural effusion (aOR, 0.25; 95% CI, 0.11–0.51; *p* < 0.001) (Figure S2).

### Causal-forest analysis of continuous BMI

All 1,165 patients contributed to the causal-forest analysis in each of the 10 imputed datasets. No patients were excluded from the primary causal-forest analysis because of missing covariate data. In the overall cohort, the orthogonalized causal forest yielded an average partial-effect estimate of −0.031 (95% CI, −0.037 to −0.025; *p* < 0.001) on the absolute PPC probability scale per 1 kg/m^2^ higher BMI. This estimate represents a model-based average partial slope across the study population and should not be interpreted as evidence that deliberately increasing an individual child’s BMI would necessarily reduce that child’s PPC risk by 3.1 percentage points (Table 3). The distribution of BMI and the corresponding continuous-exposure support diagnostics across sex, study center, and preoperative Cobb-angle strata are presented in Figure S4 and Table S8. Empirical support was more limited near the extreme tails of the BMI distribution, and estimates in these regions were therefore interpreted cautiously. Figure S3 provides an exploratory descriptive visualization of model-derived conditional partial-effect predictions across sex and preoperative Cobb-angle strata. Greater variability was observed near the extremes of the BMI distribution, where empirical support was more limited.

**Table 3.** Orthogonalized causal-forest estimate of the average partial effect of continuous BMI on PPCs in the overall cohort.

Model	Analysis population	Effect estimate	95% CI	*P*
Orthogonalized causal forest	Overall cohort	−0.031	(−0.037 to −0.025)	< 0.001

Abbreviations: BMI, body mass index; APE, average partial effect; CI, confidence interval; PPCs, postoperative pulmonary complications. BMI was modelled as a continuous exposure. The estimate is expressed on the absolute PPC probability scale per 1 kg/m² higher BMI and represents a model-based average partial slope rather than a conventional binary-treatment average treatment effect. Within each imputed dataset, the APE and its variance were obtained using average_treatment_effect(); estimates were pooled across the 10 imputed datasets using Rubin’s rules. The estimate should not be interpreted as the effect of deliberately increasing an individual patient’s BMI.

### Exploratory pathway analysis

Exploratory pathway analyses suggested small model-based indirect components through PNI (ACME, −0.0012; *p* < 0.001), intraoperative blood loss (ACME, −0.0018; *p* = 0.012), and fusion levels (ACME, −0.0011; *p* < 0.001), corresponding to model-based estimated proportions of 7.1%, 10.1%, and 5.8%, respectively. These findings suggest that nutritional-immune status and operative burden may represent potential partial pathways, but they do not establish causal mediation (Table 4).

**Table 4.** Exploratory pathway analysis of potential indirect components in the association between BMI and PPCs.

Potential pathway	Model-based indirect component (ACME)	*P* value	Remaining/direct component (ADE)	Estimated total association	Estimated proportion attributed
PNI	−0.0012	< 0.001	−0.015	−0.0170	0.071
Blood Loss	−0.0018	0.012	−0.016	−0.0178	0.101
Fusion Levels	−0.0011	< 0.001	−0.018	−0.0192	0.058

Note: Estimates are presented on the absolute PPC probability scale. The mediation framework conventionally labels the estimated quantities as the average causal mediation effect (ACME) and average direct effect (ADE); however, in this retrospective analysis, these quantities are interpreted only as model-based indirect and remaining/direct components of the observed association. The “estimated total association” represents the corresponding model-based total association. These exploratory estimates should not be interpreted as evidence of causal mediation. BMI, body mass index; PNI, Prognostic Nutritional Index; PPCs, postoperative pulmonary complications.

### Predictive value

At the predefined end-of-surgery prediction time point, the development cohort comprised 882 patients with 243 PPC events, whereas the external validation cohort comprised 283 patients with 78 events. Their baseline characteristics are presented in Tables S9 and S^10^.

**Figure 3.**
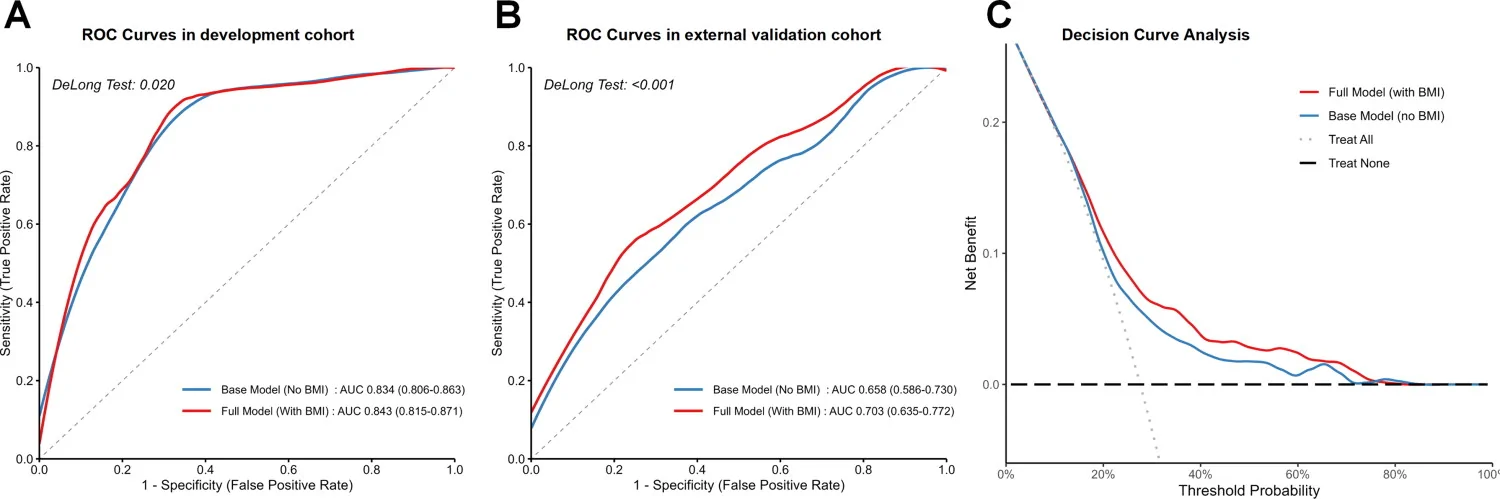
Predictive performance and decision-curve analysis of the end-of-surgery perioperative prediction models for PPCs. Receiver operating characteristic (ROC) curves and decision curve analysis (DCA) evaluating the incremental predictive value of BMI for PPCs in paediatric scoliosis surgery. (A) ROC curves in the development cohort. (B) ROC curves in the external validation cohort. The blue lines represent the base model (excluding BMI), and the red lines represent the full model (including BMI). Area under the curve (AUC) values with 95% CIs are provided; P values reflect the DeLong test for comparing the two AUCs. (C) Decision curve analysis in the external validation cohort, illustrating the clinical net benefit of the full model (red line) compared to the base model (blue line), the ‘treat-all’ strategy (grey line), and the “treat-none” strategy (horizontal black line) across a range of threshold probabilities.

In the development cohort, adding BMI to the base model produced a small increase in the AUC from 0.834 to 0.843 (*p* = 0.020) and a slight reduction in the Brier score from 0.179 to 0.177. The net reclassification improvement (NRI, −0.072; *p* = 0.340) and integrated discrimination improvement (IDI, −0.001; *p* = 0.653) were not statistically significant. In the external validation cohort, adding BMI increased the AUC from 0.658 to 0.703 (*p* < 0.001), with a reduction in the Brier score from 0.185 to 0.176. The NRI was 0.441 (*p* < 0.001), and the IDI was 0.021 (*p* < 0.001). These findings indicate a modest incremental improvement in external discrimination rather than strong predictive performance (Figure 3 and Table S11).

In the external validation cohort, the calibration intercept and slope were 0.024 (95% CI, −0.358 to 0.405) and 1.086 (95% CI, 0.739 to 1.434), respectively, for the base model, and 0.137 (95% CI, −0.237 to 0.510) and 1.190 (95% CI, 0.858 to 1.522), respectively, for the full model. Decision-curve analysis showed that the full model provided greater net benefit than the base model over selected, but not all, threshold-probability ranges (Figure 3, Figure S5, and Tables S11 and S^12^).

**Figure 4.**
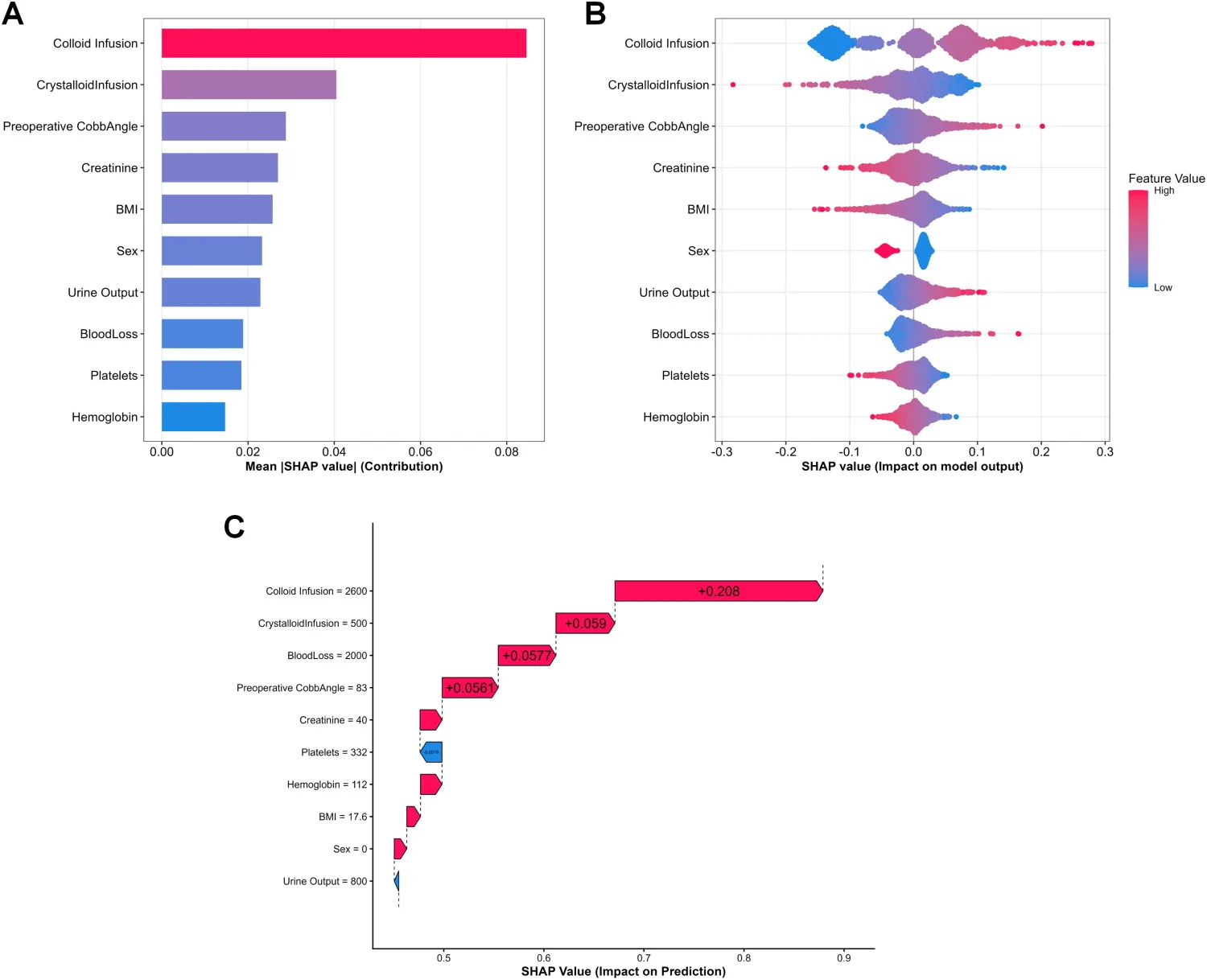
Interpretability of the full perioperative prediction model. Interpretability analysis of the full perioperative prediction model using SHapley Additive exPlanations (SHAP). (A) Global feature importance ranking based on the mean absolute SHAP values, representing the average magnitude of each variable’s contribution to the model’s overall predictions. (B) SHAP beeswarm plot illustrating the distribution of the impact of each feature on model output. Each point represents an individual patient; colour denotes the feature value (red for high, blue for low), and the x-axis position indicates the direction and extent of the impact on the predicted probability of PPCs. (C) SHAP waterfall plot providing a local explanation for a single prediction, demonstrating how individual feature values (BMI = 17.6 kg/m^2^) shift the prediction from the baseline value to the final output.

SHAP identified colloid and crystalloid infusion, preoperative Cobb angle, and BMI among the strongest contributors to predictions from the full model (Figure 4(A)). Higher colloid infusion and larger Cobb angles were associated with higher predicted PPC risk, whereas higher BMI and higher crystalloid infusion were associated with lower predicted risk in the fitted model (Figure 4(B)). The SHAP waterfall plot provides an illustrative patient-level explanation of how individual predictor values contributed to one model prediction and was not intended to support individualized clinical decision-making (Figure 4(C)).

### Sensitivity analysis

The complete-case sensitivity analysis yielded estimates consistent in direction with the primary analysis. Across all four models, BMI remained inversely associated with PPCs in the crude model (OR, 0.87; 95% CI, 0.83–0.91; *p* < 0.001), after adjustment for demographic factors (aOR, 0.88; 95% CI, 0.83–0.92; *p* < 0.001), in the primary preoperative-adjusted model (aOR, 0.88; 95% CI, 0.83–0.92; *p* < 0.001), and in the secondary exploratory perioperative-adjusted model (aOR, 0.86; 95% CI, 0.82–0.91; *p* < 0.001) (Tables S13 and S^14^).

In the supplementary analysis using preoperative body weight as the exposure, higher body weight was associated with lower odds of PPCs in the crude model (OR, 0.98; 95% CI, 0.96–0.99; *p* < 0.001) and in the model adjusted for sex, age, and study centre (aOR, 0.98; 95% CI, 0.96–0.99; *p* = 0.012). After further adjustment for preoperative laboratory variables and preoperative Cobb angle, the association was attenuated and was no longer statistically significant in Model 3 (aOR, 0.99; 95% CI, 0.97–1.00; *p* = 0.143; Table S15).

Both total intraoperative fluid volume and weight-adjusted total fluid volume were associated with PPCs in crude models. However, after adjustment for demographic and laboratory variables and preoperative Cobb angle, these associations were no longer statistically significant. The adjusted associations were not statistically significant for total fluid volume per 100 mL (aOR, 0.99; 95% CI, 0.96–1.01; *p* = 0.265) or for weight-adjusted total fluid volume per 10 mL/kg (aOR, 1.01; 95% CI, 0.93–1.09; *p* = 0.798) (Table S16).

In the sensitivity analysis based on the mutually exclusive primary PPC classification, patients whose primary documented PPC diagnosis was pleural effusion were not counted as events. The sensitivity endpoint therefore comprised 108 patients (9.27%) whose primary documented PPC diagnosis was pneumonia, atelectasis, pneumothorax, or respiratory failure. In the adjusted analysis, higher BMI remained associated with lower odds of this primary non-pleural PPC endpoint (aOR, 0.94; 95% CI, 0.91–0.97; *p* < 0.001) **(**Table S17).

Marked between-center variation was observed in the retrospectively recorded outcomes. Composite PPC frequencies were 39.86% (230/577), 27.56% (78/283), and 4.26% (13/305) in centres 1, 2, and 3, respectively. The corresponding frequencies for the primary non-pleural PPC sensitivity endpoint were 16.29% (94/577), 2.12% (6/283), and 2.62% (8/305), respectively (Table S5). Center-specific postoperative imaging proportions were unavailable.

In leave-one-centre-out analyses, the inverse association remained statistically significant after sequential exclusion of each centre (aOR range, 0.78–0.90; all *p* < 0.001; Table S18).

## Discussion

This multicentre cohort study found an inverse association between preoperative BMI and retrospectively recorded PPCs in the primary preoperative-adjusted model, with directionally consistent findings in the causal-forest analysis and most sensitivity analyses. The categorical findings suggested that the overall pattern primarily reflected the vulnerability of patients with thinness and the lower odds observed among patients with overweight, rather than a protective effect of obesity. Exploratory pathway analyses identified only small model-based indirect components. At the end-of-surgery prediction time point, adding BMI provided a modest incremental improvement in external discrimination; however, further prospective validation and clinical-impact assessment are required before clinical use.

In adult surgical populations, the ‘obesity paradox’—where overweight or mildly obese patients exhibit lower postoperative mortality and morbidity than their normal-weight or underweight peers—is well documented [6,19,20]. However, whether this phenomenon applies in paediatric surgery, particularly regarding pulmonary outcomes, has remained highly controversial. While some retrospective studies have linked low BMI to postoperative complications in paediatric surgery [21], higher BMI has been associated with surgical-site infection and greater resource utilization following spinal fusion [8]. In paediatric scoliosis, discrepant findings may reflect heterogeneous PPC definitions [14,22], differences in body-habitus classification and surgical case mix [23,24], inadequate adjustment for surgical complexity, and residual confounding. Prior studies nevertheless suggest that both obesity and thinness are associated with postoperative outcomes in paediatric populations [25–27]. Finally, because paediatric scoliosis correction surgery is concentrated in tertiary referral centres, existing studies often have limited sample sizes and restricted representativeness, potentially reducing both statistical power and generalizability.

Using an EPCO-informed retrospective PPC classification and an orthogonalized causal forest to flexibly adjust for measured preoperative covariates, our study adds to the evidence that body habitus is associated with respiratory outcomes after paediatric scoliosis correction surgery. Potential explanations include differences in nutritional reserve, immune status, body composition, and pulmonary mechanics; however, these mechanisms could not be established directly in this retrospective study [28–30]. The model-derived conditional partial-effect predictions were also examined descriptively across sex and preoperative Cobb-angle strata. Because subgroup inference was limited by unequal subgroup sizes and reduced empirical support at the extremes of the BMI distribution, these exploratory patterns were not considered evidence of effect modification [29,31].

Our study has several strengths, including its large multicentre design, the use of complementary conventional and machine-learning analyses, and the external validation of the prediction model. However, several inherent limitations must be acknowledged. First, the retrospective nature of this study precludes the complete elimination of unmeasured confounding; despite adjustment for measured covariates, the potential influence of variables such as specific surgical techniques, preoperative frailty, and unrecorded comorbidities cannot be entirely excluded. Furthermore, the causal-forest estimates depend on the assumptions of conditional exchangeability, consistency, positivity, and adequate nuisance-function estimation and therefore should not be interpreted as definitive intervention effects. Because the study period overlapped with the COVID-19 pandemic, unrecorded prior SARS-CoV-2 infection may have affected baseline pulmonary status and contributed to residual confounding [30,32]. Intensive care unit (ICU) stay, postoperative ventilation duration, and time to extubation were not consistently available across centres and could not be analysed.

BMI and BMI-Z scores are imperfect proxies for body composition because they do not distinguish fat mass from lean mass [33,34]. Because body composition was not measured, we could not determine whether variation in lean mass rather than adiposity contributed to the observed BMI-associated pattern or to its inconsistency across individual complications, particularly pneumonia, among patients with obesity. To address the potential influence of scoliosis-related height distortion on BMI estimation, we additionally performed a supplementary analysis using preoperative body weight as the exposure variable. Higher body weight showed a directionally consistent inverse association with PPCs in the unadjusted and minimally adjusted models, although this association was attenuated after more extensive adjustment. This attenuated pattern provides limited supporting evidence while highlighting that BMI is a clinically practical but imperfect proxy for body composition and nutritional status. Although the overall sample size was large, some subgroup sizes were relatively small, which may have resulted in imprecise effect estimates. In addition, the etiologic subtype of scoliosis was not consistently available in the retrospective databases, preventing stratification by idiopathic, neuromuscular, congenital, or other scoliosis subgroups. This limitation is clinically relevant because BMI-related postoperative risk patterns reported in adolescent idiopathic scoliosis [9,26] may not be directly generalizable to neuromuscular scoliosis, in which BMI categories have shown different associations with postoperative complications [35].

Although the PPC categories were informed by the EPCO framework, outcome ascertainment relied on retrospectively documented diagnoses and available imaging reports rather than prospective, standardized, or independent adjudication. Marked between-centre variation was observed in the retrospectively recorded PPC frequencies. Given this heterogeneity, we additionally performed leave-one-centre-out sensitivity analyses, in which the inverse association remained statistically significant after sequential exclusion of each centre. These findings suggest that the pooled association was not driven solely by any single centre; however, neither centre fixed-effect adjustment nor leave-one-centre-out analyses can eliminate differential outcome ascertainment. The absence of significant differences in postoperative or total hospital stay also suggests that some recorded events may have been mild, radiographically detected, or self-limited. The composite endpoint should therefore be interpreted as a retrospectively recorded and clinically heterogeneous outcome. Future prospective studies should apply standardized imaging protocols and collect pulmonary-complication severity indicators.

From a clinical perspective, BMI should be regarded primarily as a simple preoperative risk-stratification marker rather than as a direct therapeutic target. In particular, low BMI or thinness may help identify patients who warrant closer nutritional assessment, evaluation of pulmonary reserve, and intensified perioperative monitoring for PPCs. Larger prospective registries with standardized diagnostic classification are therefore needed to validate our findings.

## Conclusion

In the primary model restricted to preoperative covariates, higher preoperative BMI was associated with lower odds of PPCs among pediatric patients undergoing scoliosis correction surgery. The orthogonalized causal forest yielded a concordant inverse average partial-effect estimate under its identification assumptions, while exploratory pathway analyses accounted for only small proportions of the association. Low BMI or thinness may therefore serve as a preoperative risk marker rather than a direct therapeutic target. At the completion of surgery, adding BMI to the perioperative prediction model provided a modest incremental improvement in discrimination, but the model requires further prospective validation before clinical application.

## Supplementary Material

Supplementary Tables.docx

Supplementary Figures.pdf

## Data Availability

The datasets generated and/or analyzed during the current study are not publicly available because they contain potentially identifiable patient information. Data access requests should be directed to the corresponding author (Xiaoping Gu, xiaopinggu@nju.edu.cn).
