# Supplementary material for: Association of body mass index with postoperative pulmonary complications and potential explanatory pathways in paediatric scoliosis correction surgery: a multicentre cohort study
Source: Ann Med. 2026 Sep 17;58(1):2731716. doi: 10.1080/07853890.2026.2731716 (PMC13587573; doi:10.1080/07853890.2026.2731716)

Supplementary Figures

Figure S1 Directed Acyclic Graph of BMI and PPCs.

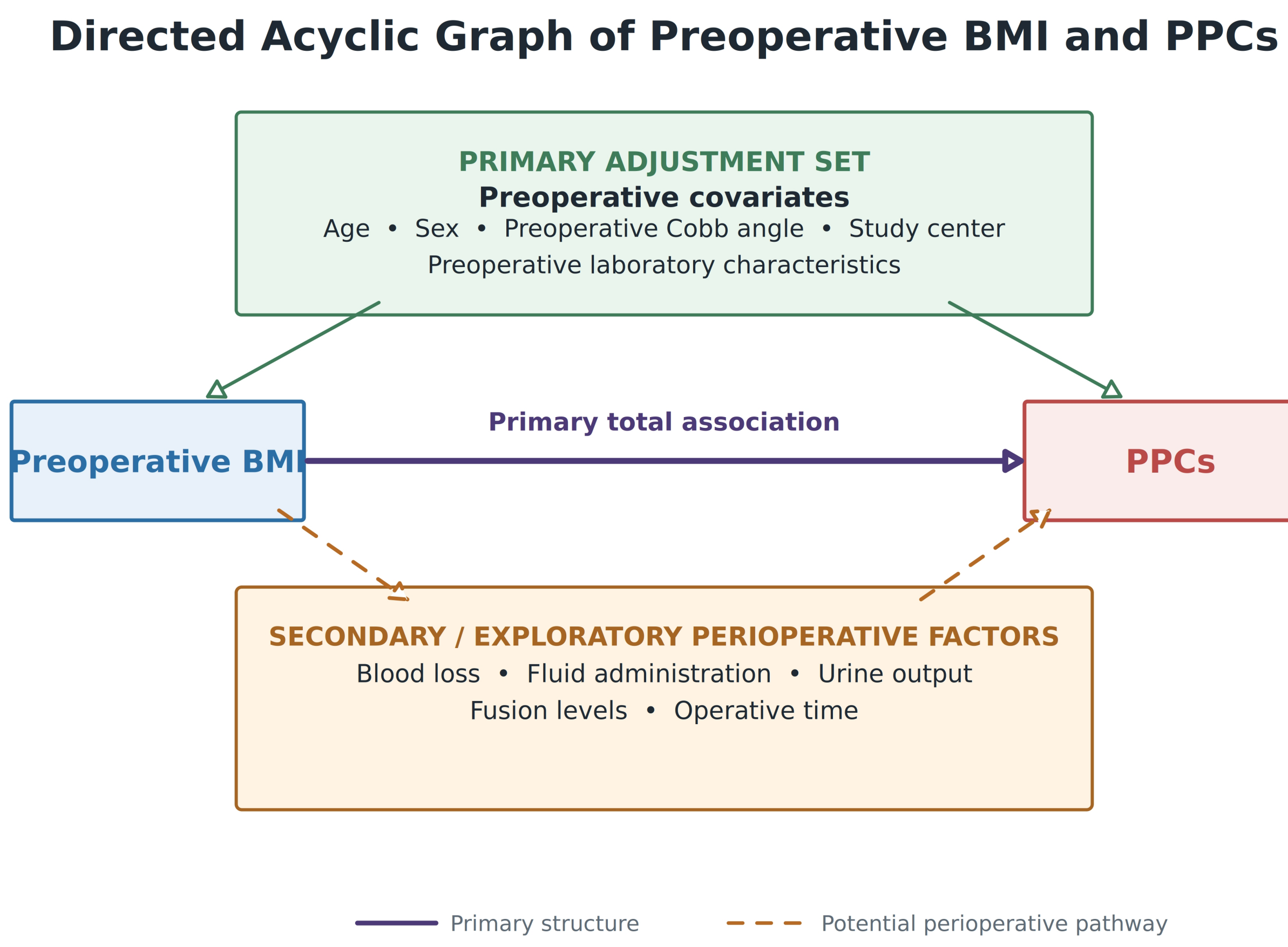

Figure S2 Forest plot of the associations between body habitus categories and the risk of specific PPCs.

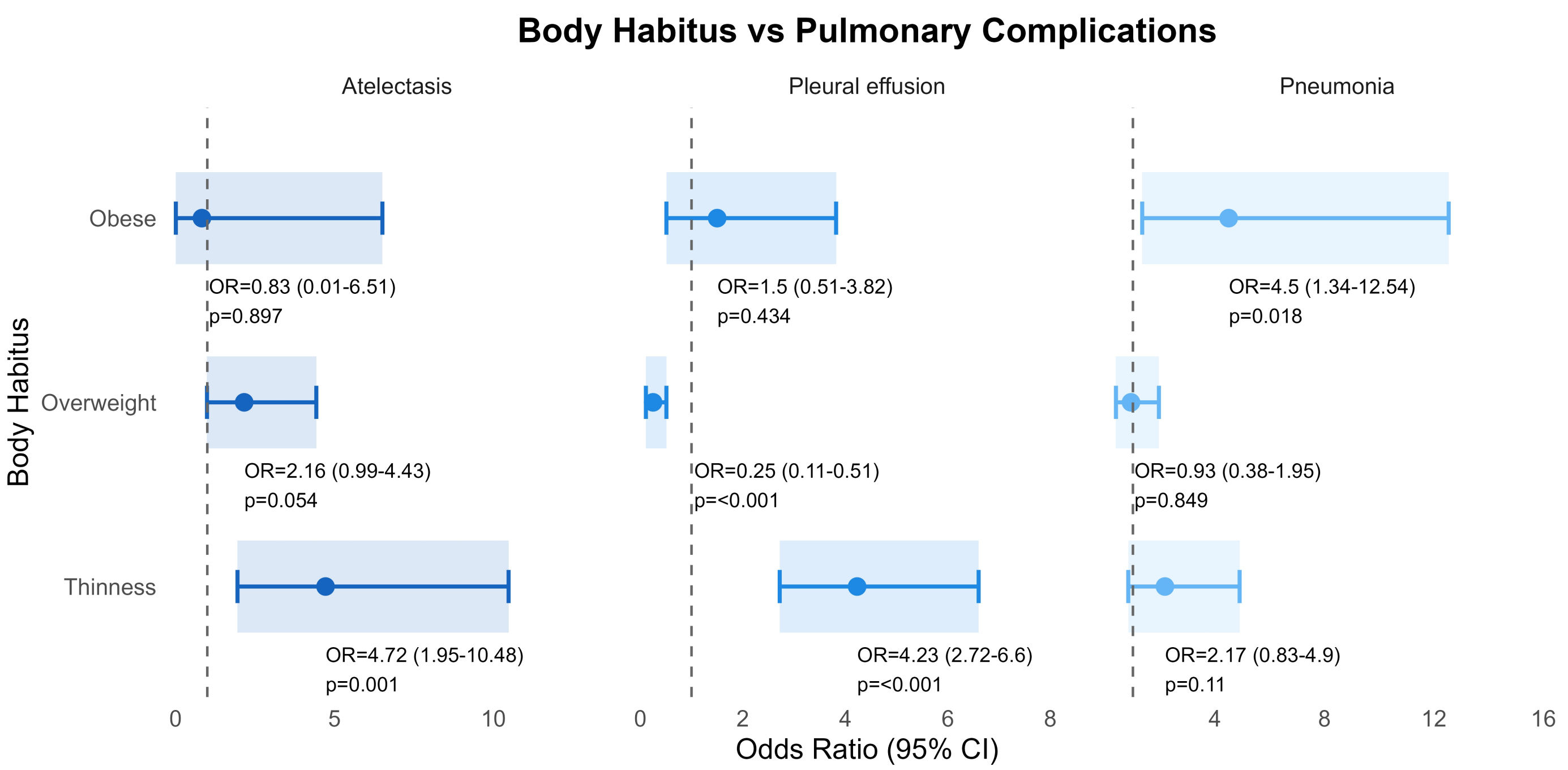

Figure S3 Exploratory descriptive variation in model-derived conditional partial-effect predictions across sex and preoperative Cobb-angle strata.

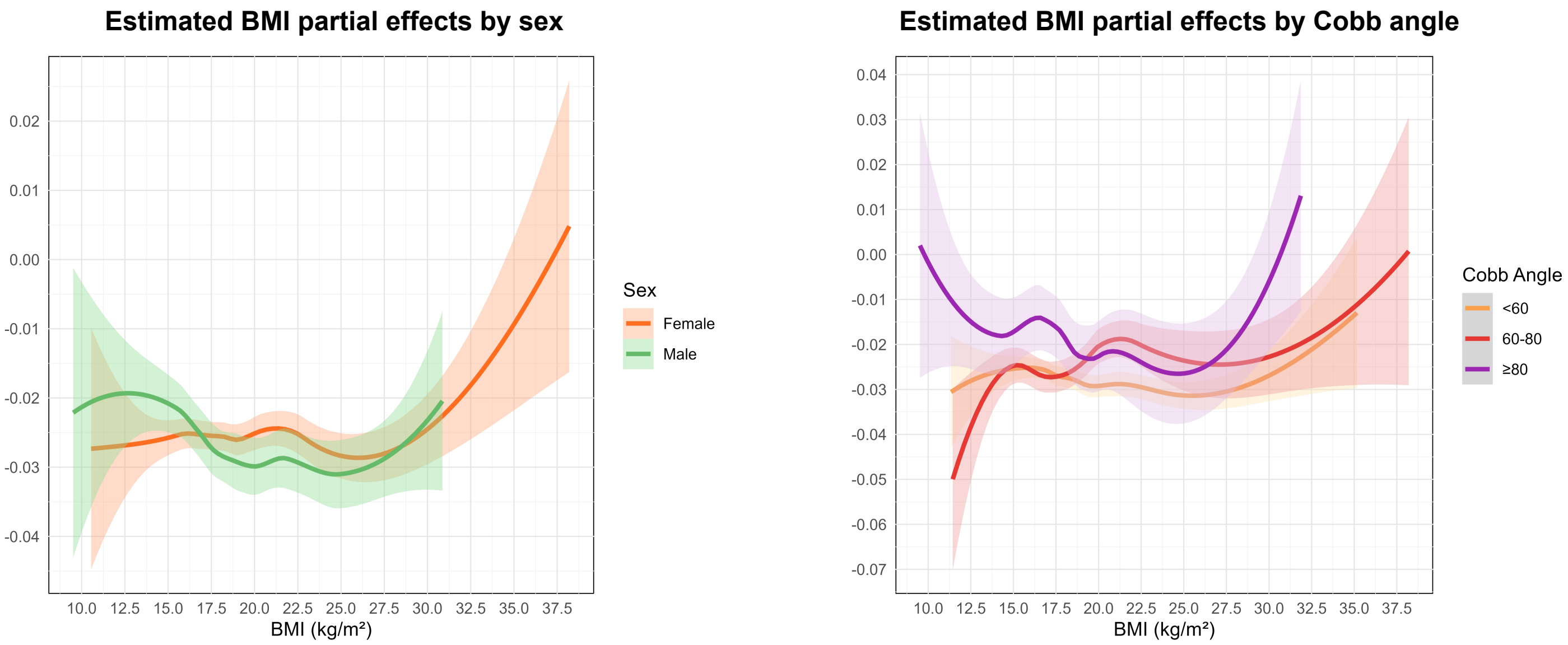

Figure S4 Empirical support of continuous BMI and distribution of estimated conditional partial effects.

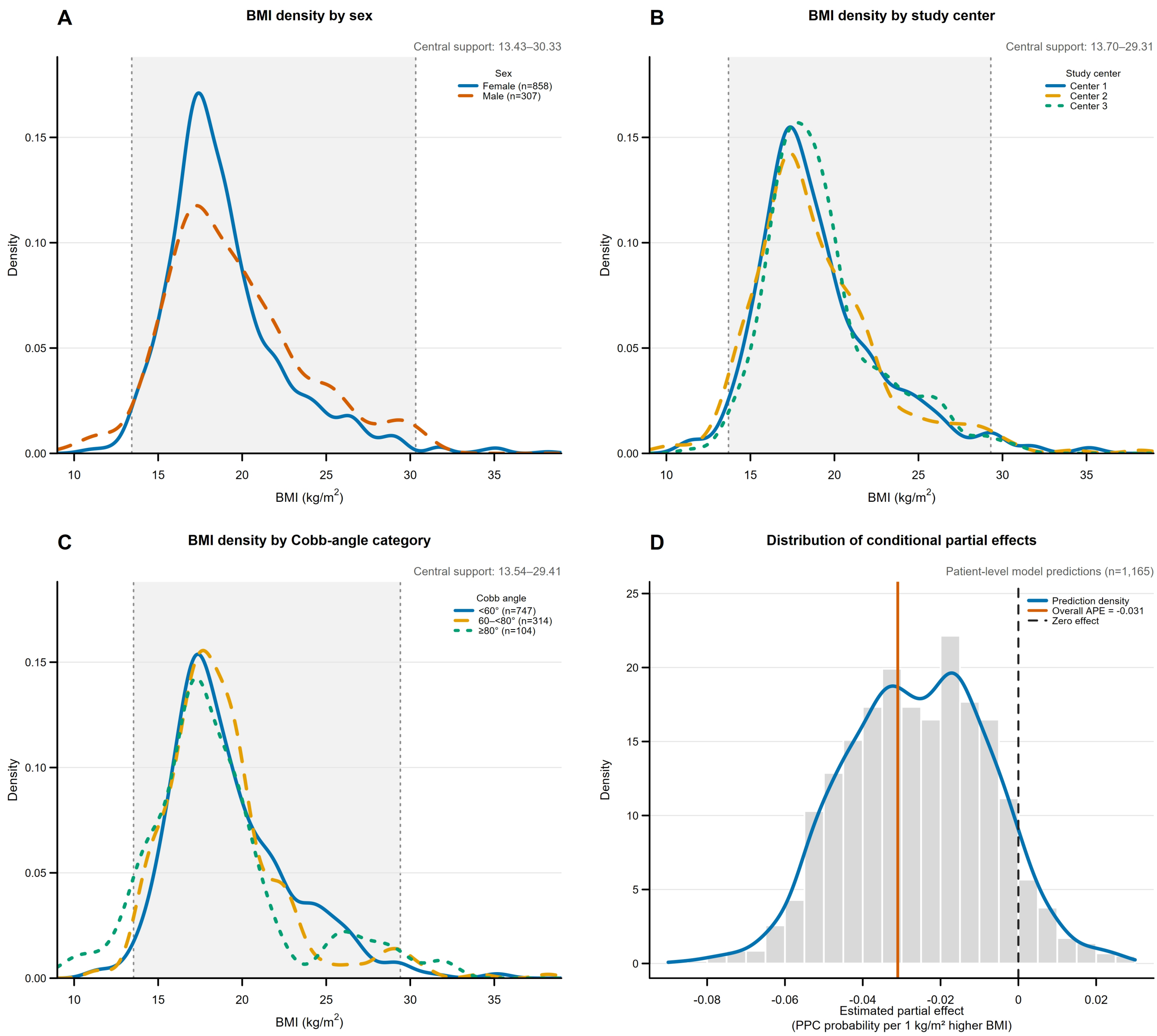

Figure S5 Calibration of the base and full models in the development and external validation cohorts.

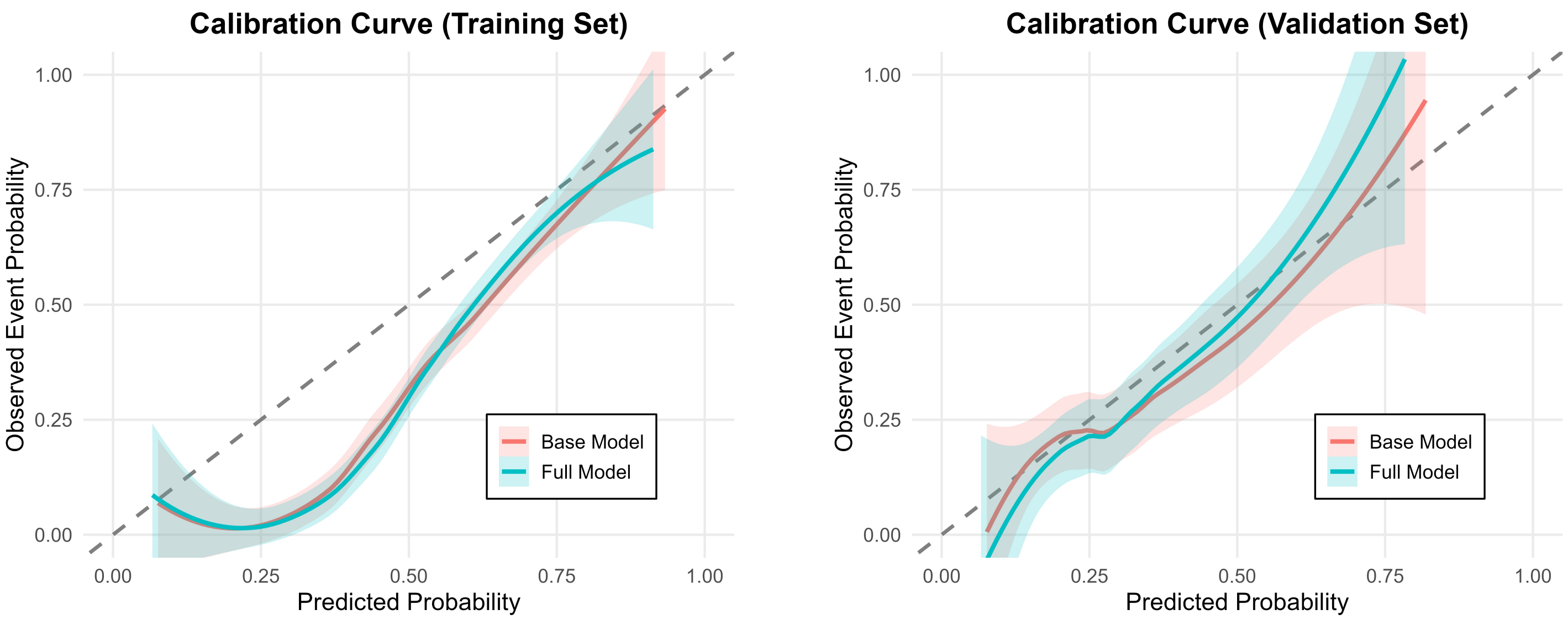

Supplement: Supplementary file 1 [file IANN_A_2731716_SM7346.pdf]
