# Supplementary material for: Association of body mass index with postoperative pulmonary complications and potential explanatory pathways in paediatric scoliosis correction surgery: a multicentre cohort study
Source: Ann Med. 2026 Sep 17;58(1):2731716. doi: 10.1080/07853890.2026.2731716 (PMC13587573; doi:10.1080/07853890.2026.2731716)
Supplement: Supplementary file 2 [file IANN_A_2731716_SM7347.docx]

**Supplementary Tables**

**Table S1 Extent of missing data for each variable.**

| **Variables** | **Total n** | **Observed, n (%)** | **Missing, n (%)** | **Missing rate (%)** | **Imputed** |
| --- | --- | --- | --- | --- | --- |
| Center | 1165 | 1165 (100.00%) | 0 (0.00%) | 0.00 | No |
| Height (cm) | 1165 | 1165 (100.00%) | 0 (0.00%) | 0.00 | No |
| Weight (kg) | 1165 | 1165 (100.00%) | 0 (0.00%) | 0.00 | No |
| BMI (kg/m²) | 1165 | 1165 (100.00%) | 0 (0.00%) | 0.00 | No |
| Sex | 1165 | 1133 (97.25%) | 32 (2.75%) | 2.75 | Yes |
| Age (years) | 1165 | 1156 (99.23%) | 9 (0.77%) | 0.77 | Yes |
| ALT (U/L) | 1165 | 1110 (95.28%) | 55 (4.72%) | 4.72 | Yes |
| AST (U/L) | 1165 | 1136 (97.51%) | 29 (2.49%) | 2.49 | Yes |
| Albumin (g/L) | 1165 | 1153 (98.97%) | 12 (1.03%) | 1.03 | Yes |
| Total bilirubin (μmol/L) | 1165 | 1136 (97.51%) | 29 (2.49%) | 2.49 | Yes |
| Creatinine (μmol/L) | 1165 | 1144 (98.20%) | 21 (1.80%) | 1.80 | Yes |
| Blood urea nitrogen | 1165 | 1142 (98.03%) | 23 (1.97%) | 1.97 | Yes |
| WBC (×10⁹/L) | 1165 | 1153 (98.97%) | 12 (1.03%) | 1.03 | Yes |
| Neutrophil count (×10⁹/L) | 1165 | 1012 (86.87%) | 153 (13.13%) | 13.13 | Yes |
| Lymphocyte count (×10⁹/L) | 1165 | 1138 (97.68%) | 27 (2.32%) | 2.32 | Yes |
| Monocyte count (×10⁹/L) | 1165 | 1073 (92.10%) | 92 (7.90%) | 7.90 | Yes |
| Hematocrit (%) | 1165 | 1147 (98.45%) | 18 (1.55%) | 1.55 | Yes |
| Hemoglobin (g/L) | 1165 | 1133 (97.25%) | 32 (2.75%) | 2.75 | Yes |
| Platelets (×10⁹/L) | 1165 | 1148 (98.54%) | 17 (1.46%) | 1.46 | Yes |
| Fibrinogen (g/L) | 1165 | 1156 (99.23%) | 9 (0.77%) | 0.77 | Yes |
| Prothrombin time (s) | 1165 | 1134 (97.34%) | 31 (2.66%) | 2.66 | Yes |
| APTT (s) | 1165 | 1118 (95.97%) | 47 (4.03%) | 4.03 | Yes |
| International normalized ratio | 1165 | 1150 (98.71%) | 15 (1.29%) | 1.29 | Yes |
| Blood loss (mL) | 1165 | 1115 (95.71%) | 50 (4.29%) | 4.29 | Yes |
| Urine output (mL) | 1165 | 1147 (98.45%) | 18 (1.55%) | 1.55 | Yes |
| Crystalloid infusion (mL) | 1165 | 1083 (92.96%) | 82 (7.04%) | 7.04 | Yes |
| Colloid infusion (mL) | 1165 | 1123 (96.39%) | 42 (3.61%) | 3.61 | Yes |
| Number of fusion levels | 1165 | 1024 (87.90%) | 141 (12.10%) | 12.10 | Yes |
| Preoperative Cobb angle (°) | 1165 | 1129 (96.91%) | 36 (3.09%) | 3.09 | Yes |
| Operative time (min) | 1165 | 1009 (86.61%) | 156 (13.39%) | 13.39 | Yes |
| Postoperative pulmonary complications, category | 1165 | 1165 (100.00%) | 0 (0.00%) | 0.00 | No |
| Postoperative pulmonary complications | 1165 | 1165 (100.00%) | 0 (0.00%) | 0.00 | No |
| Postoperative ICU stay | 1165 | 1146 (98.37%) | 19 (1.63%) | 1.63 | Yes |
| Postoperative hospital stay | 1165 | 1136 (97.51%) | 29 (2.49%) | 2.49 | Yes |
| Total hospital stay | 1165 | 1114 (95.62%) | 51 (4.38%) | 4.38 | Yes |

**Table S2 Variable-specific missingness in covariates included in the primary preoperative-adjusted model, according to study center and PPC status.**

| **Variable** | **Overall** **(N = 1,165)** | **Center 1** **(n = 577)** | **Center 2** **(n = 283)** | **Center 3** **(n = 305)** | ***P*** | **Without PPCs** **(n = 844)** | **With PPCs** **(n = 321)** | ***P*** |
| --- | --- | --- | --- | --- | --- | --- | --- | --- |
| Sex | 32 (2.75) | 14 (2.43) | 4 (1.41) | 14 (4.59) | 0.050 | 25 (2.96) | 7 (2.18) | 0.466 |
| Age (years) | 9 (0.77) | 6 (1.04) | 1 (0.35) | 2 (0.66) | 0.687 | 5 (0.59) | 4 (1.25) | 0.270 |
| ALT (U/L) | 55 (4.72) | 23 (3.99) | 19 (6.71) | 13 (4.26) | 0.189 | 38 (4.50) | 17 (5.30) | 0.568 |
| AST (U/L) | 29 (2.49) | 14 (2.43) | 6 (2.12) | 9 (2.95) | 0.804 | 24 (2.84) | 5 (1.56) | 0.208 |
| Albumin (g/L) | 12 (1.03) | 3 (0.52) | 4 (1.41) | 5 (1.64) | 0.185 | 10 (1.18) | 2 (0.62) | 0.528 |
| Creatinine (μmol/L) | 21 (1.80) | 9 (1.56) | 5 (1.77) | 7 (2.30) | 0.736 | 16 (1.90) | 5 (1.56) | 0.698 |
| WBC (×10^9^/L) | 12 (1.03) | 7 (1.21) | 2 (0.71) | 3 (0.98) | 0.929 | 8 (0.95) | 4 (1.25) | 0.746 |
| Hemoglobin (g/L) | 32 (2.75) | 14 (2.43) | 8 (2.83) | 10 (3.28) | 0.759 | 23 (2.73) | 9 (2.80) | 0.942 |
| Platelets (×10^9^/L) | 17 (1.46) | 6 (1.04) | 5 (1.77) | 6 (1.97) | 0.456 | 12 (1.42) | 5 (1.56) | 0.791 |
| Fibrinogen (g/L) | 9 (0.77) | 4 (0.69) | 2 (0.71) | 3 (0.98) | 0.908 | 8 (0.95) | 1 (0.31) | 0.458 |
| Preoperative Cobb angle (°) | 36 (3.09) | 19 (3.29) | 8 (2.83) | 9 (2.95) | 0.921 | 26 (3.08) | 10 (3.12) | 0.976 |
| **Note:** Values are missing n (%) using subgroup-specific denominators. Only variables with missing data used in the primary preoperative-adjusted model are shown. P values were obtained using Pearson's chi-square test or Fisher's exact test, as appropriate. PPCs, postoperative pulmonary complications. | | | | | | | | |

**Table S3 Model coefficients and predictor coding of the perioperative prediction models.**

| **Predictor** | **Original unit or coding** | **Base Model coefficient** | **Full Model coefficient** |
| --- | --- | --- | --- |
| Model intercept (β₀) |  | −1.2510 | −1.2514 |
| Sex | 0 = female; 1 = male | -0.281 | -0.252 |
| Age | years | 0.048 | 0.041 |
| ALT | U/L | 0.015 | 0.011 |
| AST | U/L | 0.029 | 0.022 |
| Albumin | g/L | -0.055 | -0.048 |
| Creatinine | µmol/L | -0.382 | -0.351 |
| WBC | ×10⁹/L | 0.041 | 0.032 |
| Hemoglobin | g/L | -0.101 | -0.085 |
| Platelets | ×10⁹/L | -0.118 | -0.098 |
| Fibrinogen | g/L | 0.068 | 0.059 |
| Blood Loss | mL | 0.182 | 0.151 |
| Urine Output | mL | 0.223 | 0.201 |
| Crystalloid Infusion | mL | -0.651 | -0.622 |
| Colloid Infusion | mL | 0.752 | 0.721 |
| Fusion Levels | number | 0.078 | 0.069 |
| Preoperative Cobb Angle | degrees | 0.451 | 0.422 |
| Operative Time | min | 0.091 | 0.082 |
| BMI | kg/m² | - | -0.321 |
| **Note:** Coefficients are reported on the log-odds scale. Continuous predictors were standardized using the means and standard deviations estimated in the development cohort, and the same standardization parameters were applied during external validation. Sex was coded as 0 = female and 1 = male. Predicted probability was calculated as P = 1/[1 + exp(-LP)], where LP denotes the model intercept plus the sum of each model coefficient multiplied by its corresponding standardized predictor value. The full model additionally included BMI; a dash indicates that the predictor was not included. BMI, body mass index; LP, linear predictor. | | | |

**Table S4 Distribution and Frequency of Specific Postoperative Pulmonary Complication Types.**

| **PPCs** | **n (%)** |
| --- | --- |
| Pleural Effusion | 213 (66.36%) |
| Pneumonia | 59 (18.38%) |
| Atelectasis | 43 (13.40%) |
| Pneumothorax | 4 (1.25%) |
| Respiratory Failure | 2 (0.62%) |
| Each patient was counted once in the composite PPC outcome. For the subtype distribution, patients with more than one PPC were assigned to a single mutually exclusive category according to the primary documented postoperative diagnosis. The percentages therefore sum to 100%. | |

**Table S5 Center-specific frequencies of PPCs.**

| **Study center** | **Patients, n** | **Composite PPCs, n (%)** | **Primary pleural-effusion category, n (%)** | **Primary non-pleural PPC category, n (%)** |
| --- | --- | --- | --- | --- |
| Center 1 | 577 | 230 (39.86) | 136 (23.57) | 94 (16.29) |
| Center 2 | 283 | 78 (27.56) | 72 (25.44) | 6 (2.12) |
| Center 3 | 305 | 13 (4.26) | 5 (1.64) | 8 (2.62) |
| Overall | 1,165 | 321 (27.55) | 213 (18.28) | 108 (9.27) |

**Note:** Percentages use center-specific denominators. PPC subtypes were classified into mutually exclusive categories according to the primary documented postoperative diagnosis. The primary non-pleural PPC category comprised pneumonia, atelectasis, pneumothorax, and respiratory failure. These counts do not represent all individual PPC components among patients with co-occurring diagnoses. Center-specific imaging proportions were unavailable. PPCs, postoperative pulmonary complications.

**Table S6 Preoperative multivariable logistic regression results for postoperative pulmonary complications.**

| **Variable** | **β coefficient** | **SE** | **OR (95% CI)** | ***P*** |
| --- | --- | --- | --- | --- |
| BMI, per 1 kg/m² increase | -0.139 | 0.027 | 0.870 (0.828, 0.912) | <0.001 |
| Sex | -0.894 | 0.220 | 0.409 (0.266, 0.630) | <0.001 |
| Age | -0.100 | 0.045 | 0.905 (0.828, 0.989) | 0.028 |
| ALT | -0.0002 | 0.011 | 1.000 (0.979, 1.021) | 0.987 |
| AST | 0.014 | 0.014 | 1.014 (0.987, 1.043) | 0.313 |
| Albumin | -0.0001 | 0.027 | 1.000 (0.949, 1.053) | 0.997 |
| Creatinine | -0.011 | 0.0086 | 0.989 (0.972, 1.005) | 0.180 |
| WBC | 0.035 | 0.042 | 1.036 (0.953, 1.125) | 0.410 |
| Hemoglobin | -0.0055 | 0.0078 | 0.995 (0.979, 1.010) | 0.483 |
| Platelets | -0.0030 | 0.0014 | 0.997 (0.994, 1.000) | 0.029 |
| Fibrinogen | 0.056 | 0.061 | 1.058 (0.939, 1.192) | 0.357 |
| Preoperative Cobb angle | 0.027 | 0.0064 | 1.028 (1.015, 1.041) | <0.001 |
| Study center 3 vs 1 | -2.364 | 0.446 | 0.094 (0.039, 0.226) | <0.001 |
| Study center 2 vs 1 | 0.147 | 0.300 | 1.158 (0.644, 2.084) | 0.623 |
| All continuous variables were modeled per one-unit increase on their original scales. Study center was included as a fixed effect, with center 1 as the reference category. β represents the logistic regression coefficient on the log-odds scale. OR, odds ratio; CI, confidence interval; SE, standard error. | | | | |

**Table S7** **Logistic regression analysis models showing the associations between Body Habitus and postoperative pulmonary complications.**

| **Body Habitus** | **Model1** | | **Model2** | | **Model3** | | **Model4** | |
| --- | --- | --- | --- | --- | --- | --- | --- | --- |
|  | **OR (95%CI)** | ***P*** | **OR (95%CI)** | ***P*** | **OR (95%CI)** | ***P*** | **OR (95%CI)** | ***P*** |
| Normal | Ref |  | Ref |  | Ref |  | Ref |  |
| Obese | 1.73 (0.72, 3.95) | 0.2 | 1.80 (0.72, 4.25) | 0.189 | 1.56 (0.60, 3.82) | 0.341 | 1.15 (0.39, 3.37) | 0.803 |
| Overweight | 0.55 (0.34, 0.86) | 0.012 | 0.55 (0.34, 0.87) | 0.014 | 0.57 (0.34, 0.91) | 0.022 | 0.57 (0.33, 0.97) | 0.043 |
| Thinness | 3.76 (2.50, 5.71) | <0.001 | 4.68 (3.05, 7.26) | <0.001 | 4.26 (2.74, 6.67) | <0.001 | 5.46 (3.27, 9.22) | <0.001 |
| OR: Odds Ratio, CI: Confidence Interval | | | | | | | | |
| Model1: Crude | | | | | | | | |
| Model2: Adjust: Sex, Age | | | | | | | | |
| Model3 (Primary preoperative-adjusted model): Adjust: Sex, Age, ALT, AST, Albumin, Creatinine, WBC, Hemoglobin, Platelets, Fibrinogen, Preoperative Cobb Angle | | | | | | | | |
| Model4 (secondary exploratory perioperative-adjusted model): Adjust: Sex, Age, ALT, AST, Albumin, Creatinine, WBC, Hemoglobin, Platelets, Fibrinogen, Preoperative Cobb Angle, Blood Loss, Urine Output, Crystalloid Infusion, Colloid Infusion, Fusion Levels, Operative Time | | | | | | | | |
| Models 2-4 included study center as a fixed effect in addition to the listed variables. Perioperative variables included in Model 4 were considered potential intermediate factors or markers of surgical complexity rather than conventional confounders. | | | | | | | | |

**Table S8 Distribution and empirical support of continuous BMI across prespecified covariate strata.**

| **Stratification** | **Stratum** | **n** | **Mean (SD)** | **Median (IQR)** | **Observed range** | **1st–99th percentile** | **Central common-support interval** | **Within common support, n (%)** |
| --- | --- | --- | --- | --- | --- | --- | --- | --- |
| **Overall** | All patients | 1165 | 19.10 (3.70) | 18.30 (16.79–20.71) | 9.51–38.20 | 12.40–30.41 | 12.40–30.41 | 1142 (98.0%) |
| **Sex** | Female | 858 | 18.94 (3.55) | 18.07 (16.79–20.28) | 10.55–38.20 | 13.43–30.34 | 13.43–30.33 | 841 (98.0%) |
|  | Male | 307 | 19.53 (4.07) | 18.93 (16.79–21.88) | 9.51–30.86 | 11.44–30.33 | 13.43–30.33 | 294 (95.8%) |
| **Study center** | Center 1 | 577 | 19.08 (3.76) | 18.13 (16.65–20.76) | 11.34–35.16 | 12.18–31.56 | 13.70–29.31 | 550 (95.3%) |
|  | Center 2 | 283 | 19.26 (3.37) | 18.55 (17.02–20.51) | 11.50–30.86 | 13.70–29.31 | 13.70–29.31 | 277 (97.9%) |
|  | Center 3 | 305 | 18.96 (3.89) | 18.06 (16.65–20.83) | 9.51–38.20 | 12.40–30.33 | 13.70–29.31 | 289 (94.8%) |
| **Preoperative Cobb angle** | <60° | 747 | 19.28 (3.62) | 18.36 (16.82–21.19) | 11.34–35.16 | 13.39–29.41 | 13.54–29.41 | 730 (97.7%) |
|  | 60–<80° | 314 | 18.85 (3.65) | 18.25 (16.68–20.00) | 11.44–38.20 | 13.54–30.71 | 13.54–29.41 | 302 (96.2%) |
|  | ≥80° | 104 | 18.53 (4.35) | 17.58 (16.40–19.98) | 9.51–31.87 | 10.56–31.80 | 13.54–29.41 | 95 (91.3%) |
| **Note:** Central common support was defined as the intersection of stratum-specific 1st–99th percentile BMI intervals within each stratification variable. BMI, body mass index; IQR, interquartile range; SD, standard deviation. | | | | | | | | |

**Table S9 Baseline Characteristics of the Development Cohort.**

| **Variables** | **Total (n = 882)** | **Without PPCs**  **(n = 639)** | **With PPCs**  **(n = 243)** | ***P*** |
| --- | --- | --- | --- | --- |
|  |  |  |  |  |
| BMI (kg/m²) | 18.09 (16.65, 20.80) | 18.38 (16.82, 21.37) | 17.65 (16.08, 19.46) | < 0.001 |
| Age (years) | 14.00 (13.00, 15.00) | 14.00 (13.00, 15.00) | 14.00 (12.00, 15.00) | 0.044 |
| ALT (U/L) | 10.00 (8.00, 13.30) | 10.00 (8.00, 13.00) | 10.80 (8.30, 14.20) | 0.023 |
| AST (U/L) | 17.80 (15.80, 20.00) | 17.70 (15.90, 19.40) | 18.00 (15.65, 22.10) | 0.054 |
| Albumin (g/L) | 43.00 (41.00, 44.90) | 43.00 (41.00, 45.00) | 42.20 (40.40, 44.70) | 0.003 |
| Total Bilirubin (μmol/L) | 10.10 (7.40, 13.90) | 10.10 (7.40, 13.60) | 9.70 (7.05, 14.40) | 0.976 |
| Creatinine (μmol/L) | 49.00 (42.00, 57.00) | 50.00 (44.00, 58.00) | 46.00 (40.00, 52.00) | < 0.001 |
| WBC (×10⁹/L) | 6.38 (5.48, 7.40) | 6.40 (5.49, 7.40) | 6.30 (5.40, 7.30) | 0.683 |
| Hemoglobin (g/L) | 130.50 (123.00, 140.00) | 131.00 (122.50, 141.00) | 129.00 (123.00, 136.00) | 0.002 |
| Platelets (×10⁹/L) | 257.50 (220.00, 304.00) | 259.00 (223.00, 307.00) | 252.00 (219.00, 294.00) | 0.061 |
| Fibrinogen (g/L) | 2.20 (1.90, 2.60) | 2.20 (1.90, 2.60) | 2.30 (1.90, 2.58) | 0.601 |
| Prothrombin Time (s) | 11.60 (11.10, 12.10) | 11.60 (11.15, 12.20) | 11.40 (11.00, 11.90) | < 0.001 |
| APTT (s) | 28.90 (27.20, 30.58) | 29.10 (27.35, 30.60) | 28.70 (26.40, 30.35) | 0.007 |
| Blood Loss (mL) | 600.00 (386.50, 900.00) | 500.00 (300.00, 800.00) | 800.00 (600.00, 1100.00) | < 0.001 |
| Urine Output (mL) | 875.00 (500.00, 1200.00) | 800.00 (500.00, 1100.00) | 1050.00 (700.00, 1350.00) | < 0.001 |
| Crystalloid Infusion (mL) | 1700.00 (1100.00, 2300.00) | 1800.00 (1200.00, 2400.00) | 1500.00 (1000.00, 2100.00) | < 0.001 |
| Colloid Infusion (mL) | 1000.00 (0.00, 1500.00) | 1000.00 (0.00, 1500.00) | 1500.00 (1000.00, 1600.00) | < 0.001 |
| Fusion Levels, n | 9.00 (7.00, 12.00) | 9.00 (7.00, 12.00) | 10.00 (9.00, 11.00) | 0.002 |
| Preoperative Cobb Angle (°) | 53.00 (45.00, 64.00) | 51.00 (43.00, 62.00) | 58.00 (50.00, 70.00) | < 0.001 |
| Operative Time (min) | 244.80 (195.00, 290.00) | 244.80 (187.20, 290.00) | 244.80 (201.60, 295.20) | 0.017 |
| Sex, n (%) |  |  |  | < 0.001 |
| Female children | 651 (73.81) | 451 (70.58) | 200 (82.30) |  |
| Male children | 231 (26.19) | 188 (29.42) | 43 (17.70) |  |
| Data are presented as median (IQR) for continuous variables and n (%) for categorical variables. Abbreviation: BMI: Body Mass Index, ALT: Alanine Aminotransferase, AST: Aspartate Aminotransferase, WBC: white blood cell count. | | | | |

**Table S10 Baseline Characteristics of the External Validation Cohort.**

| **Variables** | **Total (n = 283)** | **Without PPCs**  **(n = 205)** | **With PPCs**  **(n = 78)** | ***P*** |
| --- | --- | --- | --- | --- |
|  |  |  |  |  |
| BMI (kg/m^2^) | 18.55 (17.02, 20.51) | 19.03 (17.58, 21.97) | 17.21 (15.81, 18.66) | < 0.001 |
| Age (years) | 14.00 (13.00, 15.00) | 14.00 (13.00, 15.00) | 13.00 (12.00, 15.00) | 0.112 |
| ALT (U/L) | 11.00 (9.00, 14.00) | 11.00 (9.00, 14.00) | 10.07 (8.00, 13.23) | 0.343 |
| AST (U/L) | 19.00 (17.00, 22.00) | 19.00 (17.00, 22.00) | 19.00 (16.10, 21.00) | 0.664 |
| Albumin (g/L) | 46.00 (43.70, 47.65) | 46.10 (44.00, 47.80) | 45.45 (43.25, 47.50) | 0.243 |
| Total Bilirubin (μmol/L) | 11.70 (9.25, 14.70) | 11.50 (8.50, 14.70) | 12.24 (9.62, 15.33) | 0.343 |
| Creatinine (μmol/L) | 58.00 (51.00, 66.00) | 59.53 (52.00, 67.00) | 54.00 (48.00, 63.75) | 0.004 |
| WBC (×10⁹/L) | 6.30 (5.38, 7.31) | 6.40 (5.41, 7.33) | 6.27 (5.30, 7.12) | 0.618 |
| Hemoglobin (g/L) | 135.00 (128.00, 143.00) | 135.00 (127.00, 144.00) | 134.00 (128.00, 140.00) | 0.502 |
| Platelets (×10⁹/L) | 261.00 (227.00, 308.50) | 264.00 (227.00, 310.00) | 261.00 (229.25, 304.75) | 0.918 |
| Fibrinogen (g/L) | 2.48 (2.20, 2.80) | 2.46 (2.20, 2.80) | 2.60 (2.34, 2.81) | 0.215 |
| Prothrombin Time (s) | 11.70 (11.20, 12.30) | 11.70 (11.20, 12.30) | 11.60 (11.20, 12.31) | 0.681 |
| APTT (s) | 33.00 (31.05, 35.30) | 32.80 (31.00, 35.20) | 33.65 (31.15, 35.58) | 0.456 |
| Blood Loss (mL) | 500.00 (400.00, 700.00) | 500.00 (400.00, 600.00) | 640.00 (400.00, 800.00) | < 0.001 |
| Urine Output (mL) | 1000.00 (700.00, 1400.00) | 1000.00 (600.00, 1300.00) | 1000.00 (712.50, 1537.50) | 0.085 |
| Crystalloid Infusion (mL) | 2200.00 (1700.00, 2700.00) | 2150.00 (1700.00, 2650.00) | 2280.00 (1825.00, 2800.00) | 0.054 |
| Colloid Infusion (mL) | 500.00 (0.00, 500.00) | 500.00 (0.00, 500.00) | 500.00 (0.00, 500.00) | 0.032 |
| Fusion Levels, n | 10.00 (8.00, 12.00) | 10.00 (7.00, 12.00) | 11.00 (10.00, 13.00) | < 0.001 |
| Preoperative Cobb Angle (°) | 54.00 (45.00, 64.00) | 52.00 (44.00, 63.00) | 58.00 (50.00, 71.75) | <0.001 |
| Operative Time (min) | 273.60 (234.00, 332.50) | 265.00 (228.00, 316.00) | 310.50 (255.00, 359.50) | < 0.001 |
| Sex, n(%) |  |  |  | 0.074 |
| Female children | 207 (73.14) | 144 (70.24) | 63 (80.77) |  |
| Male children | 76 (26.86) | 61 (29.76) | 15 (19.23) |  |
| Data are presented as median (IQR) for continuous variables and n (%) for categorical variables. Abbreviation: BMI: Body Mass Index, ALT: Alanine Aminotransferase, AST: Aspartate Aminotransferase, WBC: white blood cell count. | | | | |

**Table S11 Predictive Value of BMI for Postoperative Pulmonary Complications.**

| **Data set** | **Model** | **AUC** | **Sensitivity** | **Specificity** | **Threshold** | **Brier** | **DeLong *P*** | **NRI** | **IDI** |
| --- | --- | --- | --- | --- | --- | --- | --- | --- | --- |
| **Development Cohort** | Base Model | 0.834 | 0.909 | 0.654 | 0.445 | 0.179 | - | - | - |
|  | Full Model | 0.843 | 0.905 | 0.687 | 0.462 | 0.177 | 0.02 | -0.072  (*P* = 0.340) | -0.001 (*P*=0.653) |
| **External Validation Cohort** | Base Model | 0.658 | 0.493 | 0.785 | 0.445 | 0.185 | - | - | - |
|  | Full Model | 0.703 | 0.531 | 0.805 | 0.462 | 0.176 | < 0.001 | 0.441 (*P* < 0.001) | 0.021 (*P* < 0.001) |
| Base Model included: Sex, Age, ALT, AST, Albumin, Creatinine, WBC, Hemoglobin, Platelets, Fibrinogen, Blood Loss, Urine Output, Crystalloid Infusion, Colloid Infusion, Fusion Levels, Preoperative Cobb Angle, Operative Time. Full Model: Base Model + BMI. Sensitivity and specificity thresholds were selected in the development cohort using the Youden index and then applied unchanged to the external validation cohort. DeLong P values compare the AUCs of the full and base models. Abbreviations: AUC, area under the receiver operating characteristic curve; BMI, body mass index; IDI, integrated discrimination improvement; NRI, net reclassification improvement; PPCs, postoperative pulmonary complications. | | | | | | | | | |

**Table S12 Calibration and decision-curve analysis of the prediction models.**

| **Data set** | **Model** | **Calibration intercept, 95% CI** | **Calibration slope, 95% CI** | **DCA threshold range with positive net benefit** |
| --- | --- | --- | --- | --- |
| **Development Cohort** | Base Model | 0.000 (-0.248, 0.248) | 1.000 (0.788, 1.212) | 0.04–0.05; 0.10–0.71 |
|  | Full Model | 0.000 (-0.235, 0.235) | 1.000 (0.804, 1.196) | 0.02–0.06; 0.08–0.73 |
| **External Validation Cohort** | Base Model | 0.024 (-0.358, 0.405) | 1.086 (0.739, 1.434) | 0.04–0.70 |
|  | Full Model | 0.137 (-0.237, 0.510) | 1.190 (0.858, 1.522) | 0.02–0.71 |
| In the validation cohort, the Full Model showed higher net benefit than the Base Model across threshold probabilities of approximately 0.02–0.22, 0.24, 0.26, 0.28–0.42, and 0.46–0.73. When restricted to thresholds at which the Full Model also outperformed both treat-all and treat-none strategies, the corresponding range was 0.02–0.22, 0.24, 0.26, 0.28–0.42, and 0.46–0.71. Development-cohort calibration estimates are apparent estimates and were not optimism-corrected; external-validation estimates were obtained in center 2. CI, confidence interval; DCA, decision-curve analysis. | | | | |

**Table S13 Baseline characteristics of patients included in the sensitivity analysis.**

| **Variables** | **Total (n = 1009)** | **Without PPCs**  **(n = 758)** | **With PPCs**  **(n = 251)** | ***P*** |
| --- | --- | --- | --- | --- |
|  |  |  |  |  |
| BMI (kg/m^2^) | 18.37 (16.79, 20.91) | 18.80 (16.99, 21.64) | 17.67 (16.01, 19.54) | < 0.001 |
| Age (years) | 14.00 (13.00, 15.00) | 14.00 (13.00, 15.00) | 14.00 (12.00, 15.00) | 0.010 |
| ALT (U/L) | 10.00 (8.00, 13.30) | 10.00 (8.00, 13.00) | 10.20 (8.00, 13.30) | 0.517 |
| AST (U/L) | 18.00 (16.00, 20.04) | 18.00 (16.00, 20.00) | 18.05 (16.00, 21.18) | 0.223 |
| Albumin (g/L) | 43.50 (41.50, 45.88) | 43.70 (41.73, 46.00) | 42.90 (41.10, 45.30) | 0.005 |
| Total Bilirubin (μmol/L) | 10.60 (7.80, 14.13) | 10.60 (7.80, 13.90) | 10.60 (7.80, 14.83) | 0.341 |
| Creatinine (μmol/L) | 51.00 (44.00, 59.00) | 52.00 (45.00, 60.00) | 49.00 (40.75, 57.00) | < 0.001 |
| WBC (×10⁹/L) | 6.38 (5.44, 7.36) | 6.39 (5.44, 7.34) | 6.34 (5.47, 7.36) | 0.663 |
| Hemoglobin (g/L) | 131.00 (123.00, 141.00) | 132.00 (123.00, 142.00) | 130.00 (123.75, 136.00) | 0.003 |
| Platelets (×10⁹/L) | 259.00 (222.00, 306.00) | 260.00 (223.25, 308.00) | 255.50 (220.00, 298.00) | 0.125 |
| Fibrinogen (g/L) | 2.32 (2.04, 2.70) | 2.30 (2.01, 2.70) | 2.40 (2.10, 2.70) | 0.022 |
| Prothrombin Time (s) | 11.60 (11.10, 12.20) | 11.70 (11.20, 12.30) | 11.40 (11.00, 12.00) | < 0.001 |
| APTT (s) | 29.60 (27.80, 31.90) | 29.70 (27.90, 31.90) | 29.30 (27.30, 32.10) | 0.228 |
| Blood Loss (mL) | 560.00 (400.00, 800.00) | 500.00 (300.00, 750.00) | 700.00 (450.00, 1000.00) | < 0.001 |
| Urine Output (mL) | 900.00 (550.00, 1250.00) | 800.00 (500.00, 1200.00) | 1000.00 (700.00, 1425.00) | < 0.001 |
| Crystalloid Infusion (mL) | 1900.00 (1400.00, 2400.00) | 1900.00 (1500.00, 2572.50) | 1750.00 (1100.00, 2260.00) | < 0.001 |
| Colloid Infusion (mL) | 500.00 (0.00, 1500.00) | 500.00 (0.00, 1000.00) | 1500.00 (500.00, 1500.00) | < 0.001 |
| Number of fusion levels | 10.00 (7.00, 12.00) | 9.00 (7.00, 12.00) | 10.00 (9.00, 12.00) | < 0.001 |
| Preoperative Cobb Angle (°) | 53.00 (45.00, 63.75) | 51.00 (43.00, 62.00) | 58.00 (49.75, 69.00) | < 0.001 |
| Operative Time (min) | 245.00 (201.60, 303.00) | 244.80 (198.00, 300.75) | 259.20 (216.00, 328.80) | < 0.001 |
| Sex, n (%) |  |  |  | < 0.001 |
| Female children | 756 (74.93) | 548 (72.30) | 208 (82.87) |  |
| Male children | 253 (25.07) | 210 (27.70) | 43 (17.13) |  |
| Data are presented as median (IQR) for continuous variables and n (%) for categorical variables. The sensitivity-analysis cohort comprised patients with complete data for all variables included in the corresponding models. | | | | |

**Table S14 Association between BMI and PPCs in sensitivity analyses using multivariable logistic regression models.**

| **Variables** | **Model1** | | **Model2** | | **Model3** | | **Model4** | |
| --- | --- | --- | --- | --- | --- | --- | --- | --- |
|  | **OR (95% CI)** | ***P*** | **OR (95% CI)** | ***P*** | **OR (95% CI)** | ***P*** | **OR (95% CI)** | ***P*** |
| BMI | 0.87 (0.83, 0.91) | < 0.001 | 0.88 (0.83, 0.92) | < 0.001 | 0.88 (0.83, 0.92) | < 0.001 | 0.86 (0.82, 0.91) | < 0.001 |
| OR: Odds Ratio, CI: Confidence Interval | | | | | | | | |
| Model1: Crude | | | | | | | | |
| Model2: Adjust: Sex, Age | | | | | | | | |
| Model3 (Primary preoperative-adjusted model): Adjust: Sex, Age, ALT, AST, Albumin, Creatinine, WBC, Hemoglobin, Platelets, Fibrinogen, Preoperative Cobb Angle | | | | | | | | |
| Model4 (secondary exploratory perioperative-adjusted model): Adjust: Sex, Age, ALT, AST, Albumin, Creatinine, WBC, Hemoglobin, Platelets, Fibrinogen, Preoperative Cobb Angle, Blood Loss, Urine Output, Crystalloid Infusion, Colloid Infusion, Fusion Levels, Operative Time | | | | | | | | |
| Models 2-4 included study center as a fixed effect in addition to the listed variables. Perioperative variables included in Model 4 were considered potential intermediate factors or markers of surgical complexity rather than conventional confounders. | | | | | | | | |

**Table S15 Association between preoperative body weight and PPCs in supplementary analyses using multivariable logistic regression models.**

| **Variables** | **Model1** | | **Model2** | | **Model3** | |
| --- | --- | --- | --- | --- | --- | --- |
|  | **OR (95%CI)** | ***P*** | **OR (95%CI)** | ***P*** | **OR (95%CI)** | ***P*** |
| Weight, per 1 kg | 0.98 (0.96, 0.99) | < 0.001 | 0.98 (0.96, 0.99) | 0.012 | 0.99 (0.97, 1.00) | 0.143 |
| OR: Odds Ratio, CI: Confidence Interval | | | | | | |
| Model1: Crude | | | | | | |
| Model2: Adjust: Sex, Age | | | | | | |
| Model3 (Primary preoperative-adjusted model): Adjust: Sex, Age, ALT, AST, Albumin, Creatinine, WBC, Hemoglobin, Platelets, Fibrinogen, Preoperative Cobb Angle | | | | | | |
| Models 2-3 included study center as a fixed effect in addition to the listed variables. | | | | | | |

**Table S16** **Association between total intraoperative fluid volume and PPCs.**

| **Variables** | **Model1** | | **Model2** | |
| --- | --- | --- | --- | --- |
|  | **OR (95%CI)** | ***P*** | **OR (95%CI)** | ***P*** |
| Total intraoperative fluid volume, per 100 mL | 1.04 (1.02, 1.05) | <0.001 | 0.99 (0.96, 1.01) | 0.265 |
| Weight-adjusted total intraoperative fluid volume, per 10 mL/kg | 1.21 (1.14, 1.28) | <0.001 | 1.01 (0.93, 1.09) | 0.798 |
| OR: Odds Ratio, CI: Confidence Interval | | | | |
| Model1: Crude | | | | |
| Model2: Adjust: Sex, Age, ALT, AST, Albumin, Creatinine, WBC, Hemoglobin, Platelets, Fibrinogen, Preoperative Cobb Angle. (Study center was included as a fixed effect in Model 2.) | | | | |
| Total intraoperative fluid volume was calculated as the sum of crystalloid and colloid infusion; weight-adjusted volume was calculated by dividing total volume by preoperative body weight. | | | | |

**Table S17 Sensitivity analysis of the association between BMI and the primary non-pleural PPC endpoint based on mutually exclusive primary documented diagnoses.**

| **Exposure** | **Events/total, n/N (%)** | **Adjusted OR (95% CI)** | ***P*** |
| --- | --- | --- | --- |
| BMI (per 1 kg/m² increase) | 108/1,165 (9.27%) | 0.94 (0.91, 0.97) | <0.001 |
| Note: The sensitivity endpoint was constructed from the mutually exclusive primary documented PPC classification. Patients whose primary documented PPC diagnosis was pleural effusion were not counted as events, whereas patients whose primary documented diagnosis was pneumonia, atelectasis, pneumothorax, or respiratory failure were counted as events. All 1,165 patients remained in the analysis. Therefore, this endpoint represents primary documented non-pleural PPC categories and does not imply that all pleural-effusion events were removed from a dataset retaining all co-occurring PPC components. The model was adjusted for sex, age, ALT, AST, albumin, creatinine, WBC, hemoglobin, platelets, fibrinogen, preoperative Cobb angle, and study center. | | | |

**Table S18 Leave-one-center-out sensitivity analysis of the association between preoperative BMI and PPCs.**

| **Analysis population** | **Patients, n** | **PPC events, n (%)** | **Adjusted OR (95% CI)** | ***P*** |
| --- | --- | --- | --- | --- |
| Full cohort | 1,165 | 321 (27.55) | 0.87 (0.83, 0.91) | <0.001 |
| Excluding center 1 | 588 | 91 (15.48) | 0.78 (0.71, 0.87) | <0.001 |
| Excluding center 2 | 882 | 243 (27.55) | 0.90 (0.85, 0.95) | <0.001 |
| Excluding center 3 | 860 | 308 (35.81) | 0.83 (0.79, 0.88) | <0.001 |
| **Note:** Adjusted for sex, age, ALT, AST, albumin, creatinine, WBC, hemoglobin, platelets, fibrinogen, preoperative Cobb angle, and center fixed effects. ORs are per 1 kg/m² higher BMI. BMI, body mass index; CI, confidence interval; OR, odds ratio; PPCs, postoperative pulmonary complications. | | | | |
